# Genome-Wide Association Study on Root Traits Under Different Growing Environments in Wheat (**Triticum aestivum** L.)

**DOI:** 10.3389/fgene.2021.646712

**Published:** 2021-06-10

**Authors:** Fengdan Xu, Shulin Chen, Xiwen Yang, Sumei Zhou, Junsen Wang, Ziliang Zhang, Yuan Huang, Miao Song, Jun Zhang, Kehui Zhan, Dexian He

**Affiliations:** ^1^Co-construction State Key Laboratory of Wheat and Maize Crop Science, Collaborative Innovation Center of Henan Grain Crops, College of Agronomy Henan Agricultural University, Henan Agricultural University, Zhengzhou, China; ^2^College of Agriculture, Henan University of Sciences and Technology, Luoyang, China

**Keywords:** wheat, root traits, yield traits, genome-wide association study (GWAS), co-localization region

## Abstract

Plant roots are critical for water and nutrient acquisition, environmental adaptation, and yield formation. Herein, 196 wheat accessions from the Huang-Huai Wheat Region of China were collected to investigate six root traits at seedling stage under three growing environments [indoor hydroponic culture (IHC), outdoor hydroponic culture (OHC), and outdoor pot culture (OPC)] and the root dry weight (RDW) under OPC at four growth stages and four yield traits in four environments. Additionally, a genome-wide association study was performed with a Wheat 660K SNP Array. The results showed that the root traits varied most under OPC, followed by those under both OHC and IHC, and root elongation under hydroponic culture was faster than that under pot culture. Root traits under OHC might help predict those under OPC. Moreover, root traits were significantly negatively correlated with grain yield (GY) and grains per spike (GPS), positively correlated with thousand-kernel weight (TKW), and weakly correlated with number of spikes per area (SPA). Twelve stable chromosomal regions associated with the root traits were detected on chromosomes 1D, 2A, 4A, 4B, 5B, 6D, and unmapped markers. Among them, a stable chromosomal interval from 737.85 to 742.00 Mb on chromosome 4A, which regulated total root length (TRL), was identified under three growing environments. Linkage disequilibrium (LD) blocks were used to identify 27 genes related to root development. Three genes *TraesCS4A02G484200, TraesCS4A02G484800*, *TraesCS4A02G493800*, and *TraesCS4A02G493900*, are involved in cell elongation and differentiation and expressed at high levels in root tissues. Another vital co-localization interval on chromosome 5B (397.72–410.88 Mb) was associated with not only RDW under OHC and OPC but also TKW.

## Introduction

As the population increases, the demand for wheat doubles approximately every 20 years; therefore, a continuous yield improvement in wheat production is an urgent issue. Xie et al. (2017) found that grain number per unit area, grain per spike, and plant biomass were lower for wheat lines with shorter root lengths, while lines with longer roots had the highest biomass and grain yield. The root-shoot ratio of plants improves access to residual moisture in deep soils under drought conditions, particularly in the mid- to late-stage (Reynolds et al., 2007). According to another study, the wheat root weight density was positively correlated with grain yield and water used efficiency (Man et al., 2015). Root system facilitates aerial growth and is very critical for yield (Fang et al., 2017), and its morphological characteristics include root length, surface area, volume, diameter, and number of root tips. Root length, surface area, and volume affect the spatial arrangement of roots underground, while root diameter is associated with the ability to penetrate strong soil and drought tolerance, and the root tip is the most active part of the root system (Kabir et al., 2015; Maccaferri et al., 2016; Bai et al., 2019). Previous studies have shown that optimizing root spatial configuration may lead to a higher absorption efficiency of water and nutrients, thus improving yield levels (Osmont et al., 2007; Bishopp and Lynch, 2015). Researchers have adopted indoor cultivation methods combined with digital imaging to determine the root traits of seedlings and elucidate the mechanisms regulating root growth and development through forward genetic strategies. For instance, 46 quantitative trait loci (QTLs) of root morphological traits had been identified on 17 of 21 chromosomes, excluding chromosomes 1D, 4D, 6B, and 6D, and 4.98–24.31% of the phenotypic variation had been explained (Liu et al., 2013). Ren et al. (2012) detected 35 QTLs based on a recombinant inbred line (RIL) population of Xiaoyan 54 × Beijing 411, and these QTLs were primarily distributed on chromosomes 2B, 2D, 4B, 6A, 6B, and 7B. Cao et al. (2014) mapped a root length QTL (*qTaLRO-B1*) flanked by *Xgwm210* and *Xbarc1138.2* on chromosome 2BS using near-isogenic lines (NILs) 178A and 178B. Furthermore, a few genes regulating wheat root morphology have been identified using reverse genetics methods, including *TaZFP34* (Chang et al., 2016), lateral organ boundaries (LOB) family member *TaMOR* (Li et al., 2016), and zinc finger protein *OsCYP2* (Cui et al., 2017).

Due to the enormous size (≈17 Gb) and polyploidy of the wheat genome, research on quantitative traits in hexaploid wheat has lagged behind research in other crops, especially for root traits. GWAS is a powerful tool for identifying loci that are significantly associated with target traits based on LD in panel of accessions. In recent years, with the gradual release of genomic sequences and genotyping by using sequencing data, GWAS has become a rapid and effective method for detecting related QTLs (Neumann et al., 2011). Many types of single-nucleotide polymorphism (SNP) arrays have been successfully developed to identify candidate regions for various traits in GWAS panels, including 9K (Suraj et al., 2014), 55K (Ye et al., 2019), 90K (Sukumaran et al., 2015; Wang et al., 2017), and 660K arrays (Chen et al., 2020).

Previous studies for root primarily involved QTL mapping using biparental populations (RIL, NIL, and doubled haploid (DH) populations) based on a few simple sequence repeat markers for plants grown in greenhouses (Ren et al., 2012; Cao et al., 2014; Ayalew et al., 2016). Thus, the molecular basis of many root traits remains unclear. Furthermore, some root traits have been reported to be closely related to yield traits since QTLs have been found to be co-localized (Atkinson et al., 2015; Xie et al., 2017). Therefore, 196 wheat accessions from the Huang-Huai Wheat Region of Xie et al., 2017). Therefore, 196 wheat accessions from the Huang-Huai Wheat Region of China, were used as a GWAS panel to identify root traits in the seedling stage from three growing environments, RDW from four growth stages under OPC, and yield traits in the field (Table 1). GWAS was conducted with the Wheat 660K SNP Array. This work may elucidate the genetic basis of wheat root traits to assist in the genetic improvement of the wheat root system.

**TABLE 1:** Plant growth traits, growing environments and growth stages in this study.

Root and yield trait	Plant growing environment and growth stage
Total length of roots (TRL)	Indoor Hydroponic culture (IHC)
Total root volume (TRV)	Outdoor Pot culture (OPC)
Average root diameter (ARD)	Outdoor Hydroponic culture (OHC)
Total root area (TRA)	Seedling stage (SS)
Number of root tips (NRT)	Wintering period (WP)
Root dry weight (RDW)	Jointing stage (JS)
Grain yield per area (GY)	Mature stage (MS)
Spikes per area (SPA)	
Grains per spike (GPS)	
Thousand-kernel weight (TKW)	

## Materials and Methods

### Plant Materials

The 196 wheat accessions used in this study included new bread lines, elite cultivars, and historical varieties from the Huang-Huai Wheat Region of China. These accessions included 158 from Henan Province, nine from Shaanxi, eight from Jiangsu, ten from Shandong, four from Hebei, four from Beijing, one from Anhui, one from Shanxi, and one land variety from Sichuan (Chinese Spring) (Supplementary Table 1). Seeds were provided by the Collaborative Innovation Center of Henan Grain Crops, Henan Agricultural University.

### Experimental Design

#### Hydroponic Culture

**FIGURE 1:**
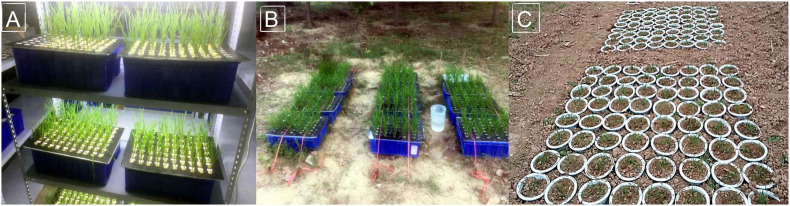
Root experiments on 196 accessions under three growing environments. **(A)** Indoor hydroponic culture (IHC); **(B)** outdoor hydroponic culture (OHC); and **(C)** outdoor pot culture (OPC).

Individuals were grown in a hydroponic system in an indoor environment (IHC). The indoor environment included day/night temperatures of 20 and 16°C, respectively, and a 16 h day length with a light intensity of 1,000 μmol⋅m^–2^⋅s^–1^ (Figure 1A). Seeds were germinated in Petri dishes and grown on germination substrate for six days with sterile water. Then, seedlings with uniform growth were transferred to plastic pots containing 18 L of nutrient solution, and fixed on a foam board with sponge (Length 5 cm; width 1 cm). The nutrient solution composition was the same as that described by Ren et al. (2012) with minor modifications. The nutrient solution was refreshed every 3 days, with the pH maintained at 6.0. Seedlings from fifty accessions were planted in one box, and 12 boxes were used for each hydroponic experiment. The 196 wheat accessions were evaluated in three replicates with two plants per replicate in each hydroponic experiment.

Another hydroponic experiment was arranged outdoors (OHC) with three replicates for three consecutive years, from October 20 to November 20 in 2016, 2017, and 2018 at Zhengzhou (34.7°N, 113.6°E), Henan, China (Figure 1B). The planting method was the same as that described above. A full box of nutrient solution was supplied daily, and a movable shelter was placed above the seedlings, especially on rainy days. All boxes for each replicate were fixed together with ropes to prevent outside interference (strong winds and, small animals).

#### Pot Experiments

All accessions were planted in pots buried in a field in 2017–2018 and 2018–2019 at Zhongmou (34.7°N, 114.0°E), Henan, China to easily extract the complete root system. The 196 wheat accessions were planted in a randomized design with two replicates for each stage. Each pot (height, 15 cm; diameter, 12 cm) was filled with 3 kg of sieved tillage soil, and the top edge of each pot was on the same plane as the ground surface (Figure 1C). The tillage soil was described as “white-sand loam” with a classification of sandy soil that contained fertilizer consisting of nitrogen at 2,295 kg⋅hm^–1^, phosphorus at 86.445 kg⋅hm^–1^ and potassium at 302.49 kg⋅hm^–1^. Before planting, seeds were germinated as described for hydroponic culture experiments. Uniformly germinating seeds were placed in each pot. Ten days after sowing, each plot was thinned to two seedlings equivalent to 1,800,000 seedlings per hectare in the field. To facilitate management, 1,568 pots representing all accessions were distributed over 20 blocks (length, 150 cm; width, 160 cm), with each block containing 80 pots. A border was planted to prevent the use of plants from around the blocks due to possible edge effects. The plants were regularly watered to 70–80% of field capacity at a 10-day interval after sowing. At jointing stage, we fertilized plants with a total nitrogen content of 3378.00 kg⋅hm^–1^, available phosphorus content of 90.00 kg⋅hm^–1^ and available potassium content of 303.75 kg⋅hm^–1^. Other field management procedures were performed in accordance with local agronomic practices. Wheat plants cultivated under OPC were as normal as those grown in the field at different growth stages, with each plant having tillers (2–5) and ears (1–3) (Supplementary Figure 1).

#### Root Phenotype Evaluation

For the hydroponic culture experiments, roots were rinsed with sterile deionized water 30 days after sowing before they were measured. In the pot experiments, the roots were removed from the soil at 30 days (seedling stage, SS), 60 days (wintering period, with a daily mean temperature < 2°C, WP), 150 days (jointing stage, 50% of main culms grew 1.5–2 cm above the ground, JS), and 220 days (mature stage, grain size was normal and water content was less than 20%, MS) after sowing, and rinsed with fresh water before being measured.

Five root system traits, including total root length (TRL), total root volume (TRV), number of root tips (NRT), total root area (TRA), and average root diameter (ARD), were scanned and analyzed with a Win-RHIZO system (Canada Regent Instruments, LA6400XL) at seedling stage. After being scanned, the roots were dried at 80°C for 48 h, and root dry weight (RDW) was measured using an analytical balance (Germany Sartorius, QUINTIX224-ICN). Only RDW was measured at WP, JS and MS under OPC conditions.

#### Field Experiment of Yield Traits

Field experiments were performed to measure yield traits in Zhengzhou (34.7°N, 113.7°E) in 2014–2015 and 2015–2016, in Shangqiu (33.4°N, 115.4°E) in 2014–2015, and in Zhumadian (33.0°N, 114.1°E) in 2014–2015. The 196 accessions were planted in a randomized block design with two replicates. Each accession was planted in one plot containing four rows with 23-cm of row spacing, and each row was 1.5 m in length with 110 seeds in a north-south orientation. Twenty adjacent spikes in the middle of each plot were randomly selected to measure number of grains per spike (GPS) and then threshed and weighted to calculate thousand-kernel weight (TKW), the middle two rows (measuring 1.0 m in length) were examined to calculate grain yield (GY) and spikes per unit area (SPA).

### Statistical Analysis

Joint variance analysis, estimation of broad-sense heritability (*H*_*B*_^2^), and BLUE values of phenotypic values obtained in each environment using in linear mixed models (LMMs) were performed using IciMapping v4.0 software (Valassi and Chierici, 2014; Lei et al., 2015). Other analyses, including descriptive statistics and correlation and canonical correlation analysis, were conducted using SAS V9.4 software.

The mean values obtained from each environment were used to analyzing the differences in root traits through a comprehensive evaluation of the spatial configuration and *t-*tests. Differences in root traits under different growing environments were compared between IHC and OHC, OHC and OPC, and IHC and OPC. Each root trait was converted into *u*-values using the standardized normal distribution method to facilitate a comprehensive evaluation (Chen et al., 2020).

### 660K SNP Genotyping, GWAS, and Prediction of Candidate Genes

All 196 accessions surveyed here were genotyped using the Wheat 660K SNP Array, which was provided by Beijing Boao Crystal Code Biotechnology Co., Ltd.^1^. Quality control and filtering of raw data were performed with a missing rate threshold of 20% and a minor allele frequency (MAF) of 5% using TASSEL v5.0 software (Bradbury et al., 2007). Chen et al. (2020) used 197 wheat accessions including the 196 wheat accessions analyzed in this study, and used same as 660K SNP Array for GWAS. Genetic diversity, population structure, and LD analyses were performed using the methods reported by Chen et al. (2020) with minor revisions. Previous reports indicated that the 196 accessions could be divided into two subpopulations, which are largely consistent with the *K*-values, principal component analysis (PCA)-matrix and quantile (Q)-matrix in the present study (Jakobsson and Rosenberg, 2007; Earl and Vonholdt, 2012). Firstly, GWAS was formally performed using five models (generalized linear models (GLMs): Q and PCA; mixed linear models (MLMs): K, PCA + K, and Q + K) to obtain an optimized model using the quantile-quantile (Q-Q) plot of each trait under IHC. The best model was considered when the actual −log_10_ (*p*-value) was closest to the expected -log_10_ (*p*) value (Liu et al., 2015). An MLM correcting for both the Q-matrix and K-matrix was confirmed to reduce the population structure and relative kinship errors. Then, we used an MLM (Q + K) to analyze associations among single environment and BLUE values for each trait with Tassel v5.0 software. The threshold for the *p*-value should be set to log_10_ (1/n) when the number of SNP markers was n. However, the Bonferroni-Holm correction for multiple testing (alpha = 0.05) was too conservative and no significant SNPs were detected. To combine the GWAS results in all of the growing environments Markers with an adjusted–log_10_ (*P*-value) ≥ 3.5 were regarded as significantly associated (Beyer et al., 2019). Lastly, Manhattan and Q-Q plots were generated using the CMplot package in R^2^.

LD and haplotype block structure were analyzed using Haploview 4.2 software to obtain physical positions of the identified stable regions. IWGSCv1.0 Chinese Spring^3^ was used to retrieve candidate genes ID and sequence located in the haplotype block. Gene sequences were used to perform a BLAST search for orthologous genes in *Arabidopsis* and *Oryza sativa* L. on the NCBI website^4^. Expression pattern of candidate genes were determined by blasting the predicted coding sequences (CDS) to WheatEXP^5^ with an *E*-value cutoff 1e-10 and covering five different wheat tissues (spikes, roots, leaves, grain, and stems) each sampled at three different developmental stages.

## Results

### Phenotypic Variations of Root Traits From Different Growing Environments

The frequencies of most root traits in plants from different growing environments were normally distributed (Supplementary Figure 2). Joint variance analysis indicated very significant genotype (variety) and genotype × environment (G × E) effects (*p* ≤ 0.01) (Table 2). Root traits under OPC exhibited the greatest variation among the growing environments, followed by those of plants grown in OHC and IHC in the seedling period. In addition, the average *u*-value for each accession was used to evaluate its root morphology under different growing environments to obtain extreme root materials (Supplementary Table 1). Under IHC, the coefficients of variation (CVs) for TRT, RDW, TRV, TRL, and TRA did not differ substantially and were higher than those of TAD. Yujiao 5, Fanmai 5 and Yandian 9433 displayed poorer root development with lower *u-*values (*u* < −1.31), and Pingan 8, Yunong 416 and Luomai 21 exhibited better root development with higher *u*-values (*u* > 1.34). Under OHC, the CVs for all root traits ranged from 5.57 to 17.21%, and the heritability (*H*_*B*_^2^) ranged from 45.86 to 63.16%. Yumai 2, Yumai 8, and Luomai 23 had poorer root development with lower *u-*values (*u* < −1.22), and Sumai 3, Yake 028 and Nanda 2419 had better root development with higher *u*-values (*u* > 1.47). Under OPC, the CVs for all root traits ranged from 8.80 to 38.23%, and *H*_*B*_^2^ ranged from 42.95 to 51.80%. Kaimai 18, Zhongchuang 805 and San 160 had poorer root development with lower *u-*values (*u* < −1.22), and Zhengpinmai 8, Luohan 6 and Yanke 028 had better root development with higher *u*-values (*u* > 1.47).

**TABLE 2:** Phenotypic variation and heritability for the root traits of the 196 wheat accessions under different growing environments.

	Traits	Range	mean	CV (%)	F (G)	F (G*E)	*H*_*B*_^2^ (%)
IHC	TRL (cm)	310.3–806.4	524.8 ± 57.3	22.6	4.4**	−	70.66
	TRA (cm^2^)	40.2–125.9	77.4 ± 16.8	23.3	5.3**	−	66.81
	ARD (mm)	0.39–0.62	0.47 ± 0.03	8.3	8.4**	−	78.77
	TRV (cm^3^)	0.4–1.6	0.90 ± 0.60	26.8	3.7**	−	66.45
	NRT (count)	196.3–847.0	471.1 ± 15.6	31.2	8.6**	−	79.19
	RDW (mg)	9.8–37.5	21.7 ± 27.6	27.6	3.4**	−	54.24
OHC	TRL (cm)	564.7–1271.3	833.3 ± 14.2	14.2	11.4**	4.6**	63.2
	TRA (cm^2^)	77.5–191.1	112.0 ± 17.7	14.4	9.2**	4.9**	59.2
	ARD (mm)	0.32–0.52	0.43 ± 0.1	5.8	7.1**	6.1**	45.9
	TRV (cm^3^)	0.6–2.6	1.6 ± 1.3	17.0	6.1**	4.7**	50.8
	NRT (count)	335.8–923.7	606.5 ± 37.5	17.2	9.1**	5.0**	53.9
	RDW (mg)	25.2–53.8	37.8 ± 12.3	12.3	81.7**	72.7**	54.0
OPC-SS	TRL (cm)	224.0–1008.0	524.6 ± 48.9	27.0	7.1**	6.8**	48.0
	TRA (cm^2^)	44.1–168.8	95.3 ± 17.3	24.4	6.7**	5.8**	49.9
	ARD (mm)	0.42–0.80	0.58 ± 0.1	8.1	5.9**	6.4**	43.0
	TRV (cm^3^)	0.6–3.0	1.5 ± 0.4	28.3	6.6**	5.1**	51.8
	NRT (count)	214.3–1198.8	589.2 ± 45.6	38.2	8.3**	8.1**	47.1
	RDW (mg)	22.6–71.1	44.0 ± 24.4	24.4	131.2**	115.7**	51.0
OPC-WP	RDW (mg)	72.5–185.0	120.0 ± 19.1	19.1	243.6**	200.5**	55.9
OPC-JS	RDW (mg)	200.1–572.8	383.0 ± 45.7	22.7	7.4**	5.1**	57.3
OPC-MS	RDW (mg)	150.2–922.9	448.5 ± 34.8	34.8	72.3**	57.8**	55.0

TRL, total root length; TRV, total root volume; NRT, number of root tips; TRA, total root area; ARD, average root diameter; RDW, root dry weight; IHC, indoor hydroponic culture; OHC, outdoor hydroponic culture; OPC, outdoor pot culture; SS, seedling stage; WP, wintering period; JS, jointing stage; MS, mature stage; G, genotype; E, environment; H_B_^2^, broad sense heritability;−, indicated no variance component; **, Significant at p ≤ 0.01.

As shown in Table 2, the RDWs for wheat in pot experiments increased non-linearly over time, ranging from 44.0 (seedling) to 448.5 mg (mature), and the increase at jointing stage was the largest. In terms of individual accession, Zhengpinmai 8, Luohan 6 and Yanke 028 had higher RDWs at seedling stage, Zhoumai 13, Zhengyumai 043 and Chinese Spring had higher RDWs at wintering period, Zhengmai 379, Yunong 9901 and Jinmai 47 had higher RDWs at jointing stage, and Luohan 3, Zhengyumai 043 and Yumai 51 had higher RDWs at mature stage. By contrast, Kaimai 18, Zhongchuang 805 and San 160 had lower RDWs at seedling stage, Pu 2056, San 160 and Jinan 17 had lower RDWs at wintering period, Luomai 24, Yumai 52 and Fanmai 11 had lower RDWs at jointing stage, and Yunnong 949, Luomai 24 and Yujiao 5 had lower RDWs at mature stage. In summary, some varieties developed rapidly at earlier stages, while others developed rapidly at later stages.

### Differences in Root Traits of Wheat From Different Growing Environments

Large variations in the same root traits were found among different growing environments (Supplementary Table 2). Compared to IHC, TRL, TRA, TRV, NRT, and RDW under OHC had a very significant increase but not ARD. Under OPC, TRA, ARD, TRV, NRT, and RDW had a very significant increase, but not TRL. Compared to OHC, TRL, TRA, TRV, and NRT under OPC were reduced very significantly, while ARD and RDW were very significantly increased. Root development in wheat cultivated under IHC was slower than wheat cultivated under OHC and OPC, and root elongation under hydroponic culture was faster than that under pot culture. Compared with that of IHC, root system under OPC showed a higher canonical correlation with that under OHC, and simple correlation coefficients of root traits except for TRL, were very significant between OPC and OHC and larger than those between OPC and IHC (Table 3). None of the correlation coefficients of RDW between seedling stage under IHC and each stage under OPC were significant. However, RDW at seedling stage under OHC exhibited a very significant correlation with that of wintering period under OPC, and RDW at seedling stage under OPC had a very significant correlation with that of wintering period and mature stage under OPC (Table 4). Based on these results, root traits under OHC might provide a good prediction for those under OPC.

**TABLE 3:** Correlation coefficients of root traits between the growing environments.

Traits	IHC-vs-OHC	IHC-vs-OPC	OHC-vs-OPC
TRL	0.335**	0.059	0.292**
TRA	0.256**	0.066	0.377***
ARD	0.189*	0.063	0.093
TRV	0.162*	0.103	0.337***
NRT	0.271**	0.078	0.323***
RDW	0.124	0.016	0.378***
*V*	0.421	0.385	0.515

V, Canonical correlation coefficient; *, Significant at p ≤ 0.05; **, Significant at p ≤ 0.01; ***, Significant at p < 0.001.

**TABLE 4:** Correlation coefficients of RDW between SS and other stages.

Environments	OPC		
	WP	JS	MS
SS under IHC	–0.008	–0.068	0.108
SS under OHC	0.185**	–0.036	0.128
SS under OPC	0.335**	0.130	0.168*

*, Significant at p ≤ 0.05; **, Significant at p ≤ 0.01.

### Correlations Between Root Traits

Correlations between root traits were identical in the different growing environments (Supplementary Table 3). Except for ARD, very significant positive correlations were observed between other root traits. Among them, the correlation coefficients between TRL and TRA and between TRA and TRV were very large (approximately 0.9), and the others ranged from 0.367 to 0.834. ARD had a lower correlation with TRL, NRT, and RDW, and a very significant positive correlation with TRV.

### Correlations of Root Traits With Yield Traits

Most root traits at seedling stage under different growing environments were negatively correlated with GY, SPA, and GPS, and positively correlated with TKW. Specifically, NRT under three growing environments was significantly negatively correlated with GY. ARD and TRA under OHC were significantly negatively correlated with GPS, and RDW under OPC was also significantly negatively correlated with GPS. TRA and TRV under three growing environments were significantly positively correlated with TKW. Based on the canonical correlation analysis indicated that root traits under OHC and OPC had closer relationships with yield traits than those under IHC. Correlations of RDW at four growth stages under OPC with yield traits showed large differences. RDW at seedling stage and wintering period was significantly positively correlated with TKW, and all correlation coefficients between RDW and grain traits were low at jointing and mature stages (Table 5).

**TABLE 5:** Correlation coefficients between root traits and yield traits.

Environments	Traits	SPA	GPS	TKW	GY
IHC	TRL	0.016	–0.115	0.190**	–0.02
	TRA	–0.015	–0.113	0.194**	–0.033
	ARD	–0.084	0.009	0.047	–0.023
	TRV	–0.032	–0.104	0.175*	–0.038
	NRT	0.029	0.006	–0.067	−0.189**
	RDW	–0.066	–0.059	0.078	–0.063
	*V*	0.294			
OHC	TRL	–0.019	–0.09	0.178*	–0.104
	TRA	0.031	–0.137	0.202**	–0.116
	ARD	0.092	−0.145*	0.037	0.065
	TRV	0.064	−0.15*	0.199**	–0.088
	NRT	–0.104	–0.097	0.123	−0.183*
	RDW	–0.078	–0.061	0.134	–0.124
	*V*	0.407***			
OPC	TRL	0.009	–0.115	0.135	−0.146*
	TRA	–0.033	–0.128	0.198**	−0.157*
	ARD	–0.048	–0.039	0.040	–0.052
	TRV	–0.063	–0.115	0.213**	–0.123
	NRT	–0.017	–0.070	0.055	−0.150*
	RDW	–0.039	−0.141*	0.158*	–0.138
	*V*	0.406***			
WP at OPC	RDW	–0.069	–0.04	0.163*	–0.009
JS at OPC	RDW	–0.039	–0.02	–0.005	–0.037
MS at OPC	RDW	–0.008	0.108	–0.074	–0.066

GY, grain yield per area; SPA, spikes per area; GPS, grains per spike; TKW, thousand- kernel weight. *, Significant at p ≤ 0.05; **, Significant at p ≤ 0.01; ***, Significant at p ≤ 0.001.

### GWAS of Wheat Root Traits

After filtering, 390,136 SNP markers were available for GWAS with the optimal mixed linear model of the K + Q matrix. The GWAS panel was comprised of two subpopulations by STRUCTURE software and PCA. The LD decay distance was 15, 10, and 20 Mb in the A, B, D subgenome by Chen et al. (2020) (Supplementary Figure 3). A total of 1,329 significantly associated loci were detected for root traits from different environments, and distributed across all 21 chromosomes with an *R*^2^ range of 6.49–18.01% (Supplementary Table 4). In view of their respective growing environments, OHC was associated with the most SNP loci, followed by OPC and IHC. Using BLUE values of root traits, 484 SNPs were detected, with an *R*^2^ range of 6.38–19.43%. SNP loci of NRT were the most frequently identified, followed by those of TRL, ARD, TRV, TRA, and RDW (Supplementary Table 4 and Supplementary Figure 4).

**FIGURE 2:**
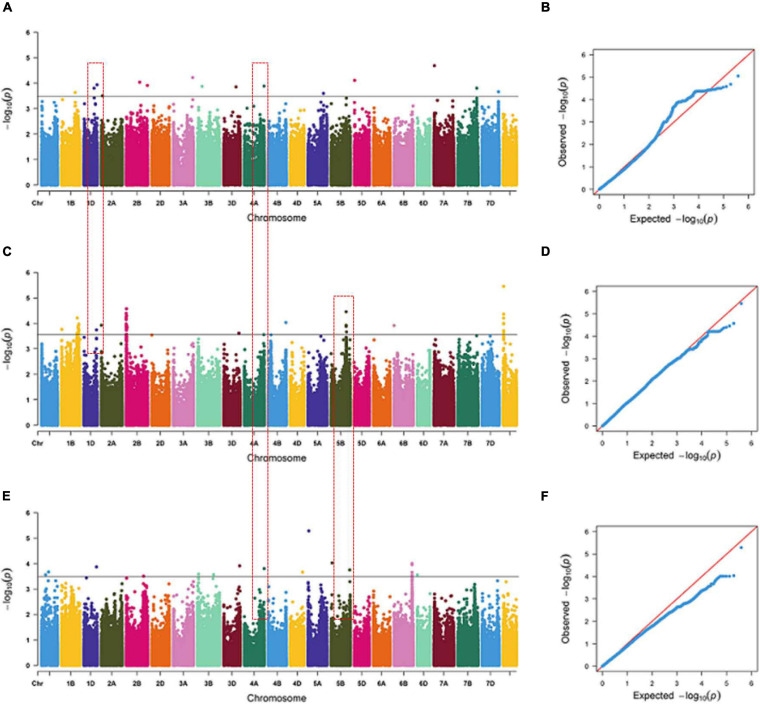
Manhattan and Q-Q plots for total root length (TRL) under indoor hydroponic culture **(A,B)**, outdoor hydroponic culture **(C,D)**, and outdoor pot culture **(E,F)**. The chromosomes on the *x*-axis and –log_10_ (*p*) values on the *y*-axis were all based on BLUE values. The gray line indicates the threshold of significance at –log_10_ (*p*) = 3.5. The red text box represents the co-localization region among different growing environments.

Some SNPs for the same trait were different, but they were detected from two or three growing environments and were located at close physical intervals. Taking 10 Mb of LD decay distance as a confidence interval (Liu et al., 2017; Chen et al., 2020), a total of 12 co-localization regions were detected from two or more growing environments, which distributed on chromosomes 1D, 2A, 4A, 4B, 5B, 6D, and unmapped markers, respectively. Among them, three co-localization regions were found for TRL, two for TRA, two for TAD, one for TRV, three for NRT, and one for RDW (Table 6). Three co-localization regions associated with TRL were located on chromosomes 1D, 4A, and 5B, explaining 9.10–10.22% of the phenotypic variation (Figure 2 and Supplementary Table 5). In particular, one co-localization region for TRL was found within the 737.85–742.00 Mb on chromosome 4A with 1 SNP each in IHC, OHC, and OPC. Two co-localization regions on chromosomes 1D and 4B for TRA were detected simultaneously in two growing environments, explaining 7.28–10.33% of the phenotypic variation. Two co-localization regions on chromosomes 5B and 6D for TAD were detected simultaneously in two growing environments, explaining 8.37–9.15% of the phenotypic variation. One co-localization region for TRV in the 543.09–547.81 Mb region on chromosome 5D, with 3 SNPs in IHC and 60 SNPs in OPC, explaining 7.04–11.82% of the phenotypic variation. Three co-localization regions on chromosomes 2A, 5B and unmapped markers for NRT were detected simultaneously in two growing environments, explaining 7.08–10.45% of the phenotypic variation. One co-localization region for RDW was found in the 397.72–410.88 Mb region on chromosome 5B, with 1 SNP in OHC and 5 SNPs in OPC, explaining 6.88–10.30% of the phenotypic variation. Of the 12 co-localization regions for root traits identified, QTL on chromosomes 1D, 4B, and 5D were reported previously (Ma et al., 2008; Kabir et al., 2015; Xie et al., 2017), while nine co-localization regions were first detected here.

**TABLE 6:** Summary of co-localization regions for root traits by GWAS.

Traits	SNP marker (Mb)		IHC	OHC	OPC
TRL	1D	464.37–465.44	2	4	
	4A	737.85–742.00	1	1	1
	5B	696.34–701.69		1	1
TRA	1D	484.26–486.74	1	2	
	4B	657.45–658.78		1	1
TAD	5B	652.10–665.20	1		1
	6D	463.54–472.27	1	12	
TRV	5D	543.09–547.80	3	60	
NRT	2A	769.56–771.34	2	1	
	5B	571.25–572.14	1	1	
	Un	71.06–77.40	1	1	
RDW	5B	397.72–410.88		1	5

*Un, unmapped markers.*

Because the root traits were closely associated with one another, some SNPs were simultaneously detected in multiple traits. For example, 9, 29, and 21 SNPs were significantly associated with two or more root traits under IHC, OHC, and OPC, respectively, and they were distributed on all chromosomes except for 1A. Moreover, five SNPs associated with three root traits were detected under OHC, and only one was found under OPC (Supplementary Table 6).

### Haploblock Analysis and Candidate Genes of TRL

**FIGURE 3:**
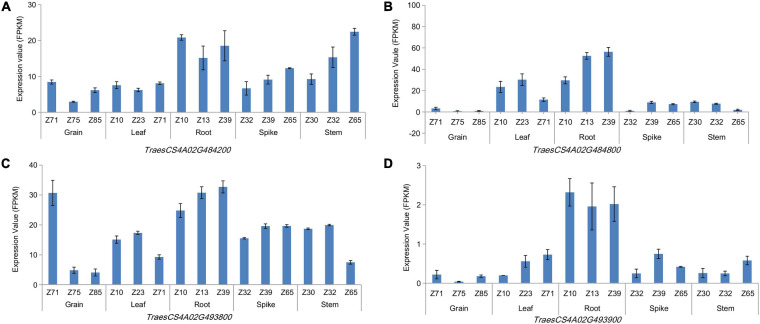
Expression patterns of genes in different wheat organs at three growing stages. *TraesCS4A02G484800* **(A)**, *Trae*sCS4A02G494200 **(B)**, TraesCS4A02G 493800 **(C)**, and TraesCS4A02G493900 **(D)**.

LD and haploblock analyses were performed for the co-localization interval 737.85–742.00 Mb on chromosome 4A, which was associated with TRL across three growing environments. And accessions with superior alleles at three growing environments showed longer TRL than others. At *r*^2^ = 0.1, two blocks were clearly separated along this 5-Mb region (Supplementary Figure 5). Further analysis showed that these two blocks were located at the physical positions 738.86–739.51 and 742.17–742.33 Mb, which contained 18 and nine genes, respectively. Then, 24 and eight orthologous genes in other species were identified in *Oryza sativa* L. and *Arabidopsis*, respectively (Supplementary Table 7). These genes encode proteins involved in growth development, carbon metabolism, energy metabolism, and plant resistance. Among them, *TraesCS4A02G484200, TraesCS4A02G484800*, *TraesCS4A02G493800*, and *TraesCS4A02G493900* showed high expression levels in roots at three growth stages (Figure 3 and Supplementary Table 8). *TraesCS4A02G484200* encodes a yippee domain protein, *TraesCS4A02G493900* encodes a member of the leucine-rich repeat domain superfamily, *TraesCS4A02G493800* encodes a member of the F-box-like domain superfamily, and *TraesCS4A02G484800* encodes the glycoside hydrolase family 32 protein. These genes might be involved in regulating root growth and development.

## Discussion

### Influence of Different Growing Environments on Wheat Root Development

The methods used to acquire root system data for crops have always been a focus in root studies. Currently, approaches are primarily divided into two categories: field and glasshouse identification (Neumann et al., 2009). Lopes and Reynolds (2010) used soil core sampling in the field to confirm a relationship between deep roots and yield across genetic differences in eight wheat sister lines under drought stress. Li et al. (2019) showed that deep-rooted accessions had lower canopy temperatures and higher grain yields than those with shallow roots as indicated by soil column testing. Field identification is labor intensive and time consuming, so it is not suitable for large-population root identification. Researchers have adopted hydroponic culture (Ayalew et al., 2016), gel-filled chambers (Christopher et al., 2013), germination bags (Robertson et al., 1979), and pots (Cao et al., 2014), to identify the seedling root traits in an artificial climate chamber and to find some QTLs or genes important for root development to overcome these challenges. Glasshouse identification may not be reflective of root development in a complex field environment, and it is difficult to apply to the study of root system in growth stages (Fang et al., 2009). Bai et al. (2019) reported a weak that correlation between the laboratory-based root length and field-based root depth across 2 years.

In this study, compared with IHC and OHC, root traits detection under OPC had a higher cost with respect to labor or time, and this system easily caused greater damage to the root system. Root traits under OHC had a very significant correlation with that under OPC and might provide them a good prediction. Considering the integrity and feasibility of root trait collection, hydroponic experiments in an outdoor or climate chamber with a suitable growing environment can be better used to identify root system in a large population.

### Relationships Between Root Traits and Yield Traits in Wheat

In wheat, the root system consists of seminal and nodal roots, seminal roots primarily absorb water and nutrients from deep soil, and nodal roots are the primary component of root system after the three-leaf stage (Osmont et al., 2007; Steinemann et al., 2015). An excessive root mass not only prolongs the growth period of a crop but also leads to strong competition for effective assimilation within plant (limited sources) (González et al., 2011). Passioura (1983) showed that an excessive outgrowth of roots caused inefficient carbohydrate consumption and yield loss. However, some studies revealed that seminal root number and TRL at seedling stage were positively correlated with GY (Maccaferri et al., 2016; Zheng et al., 2019), but negatively correlated or not correlated with TKW (Neumann et al., 2009). Atta et al. (2013) found that root traits had weak positive correlations with GPS, SPA, GY, and water use efficiency. Reducing nodal root mass can improve water use efficiency after flowering and thus increase harvest index (Ma et al., 2008). In this study, root traits were negatively correlated with SPA, GPS, and GY, and positively correlated with TKW. In most previous studies, the culture cycle was 10–14 days and the roots only consisted of seminal roots without nodal roots, but in our study, the culture cycle was 30 days after sowing with 2–3 nodal roots. Culture time of root system may be the main reason for the different results.

According to previous studies, the root system was closely related to yield from a genetic perspective. Maccaferri et al. (2016) found that some QTLs that not only improved yield traits (GY and TKW) but also increased root number and TRL in tetraploid wheat. Zheng et al. (2019) also found that *QTrl.saw-2D.2* located on chromosome 2D was a major QTL controlling TRL, and was also associated with TKW and kernel number per spike. Atkinson et al. (2015) detected two QTLs on chromosomes 2B and 7Dthat simultaneously controlled root traits at seedling stage, GY and nitrogen uptake. In rice, a QTL associated with an increased root length of 9.6 cm was introduced into a dryland variety, which increased GY by 200 kg⋅hm^–2^ (Steele et al., 2013).

**FIGURE 4:**
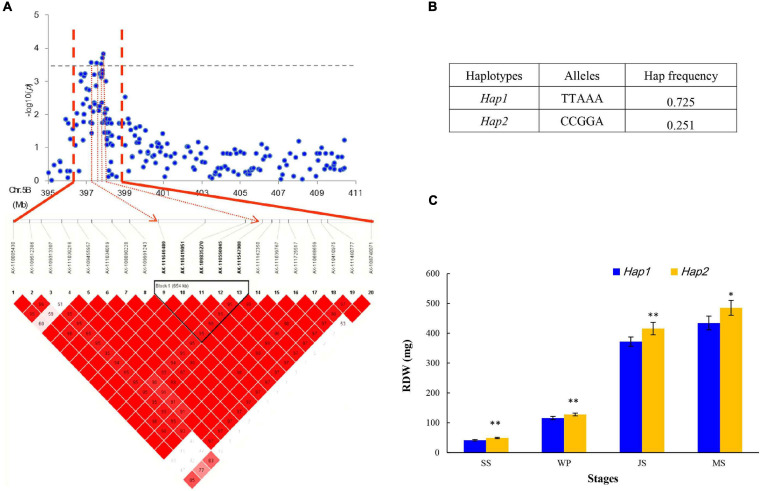
Haplotype analysis of SNPs in the stable region at 397.72–410.88 Mb on chromosome 5B. **(A)** Local Manhattan plot (top) and LD heatmap (bottom) surrounding the peak on chromosome 5B; **(B)** two haplotypes with different alleles; and **(C)** phenotypic effects of different haplotypes.

In this study, five SNPs, namely, *AX-111649489, AX-110419051, AX-109835270, AX-110550045*, and *AX-111547988* on chromosome 5B from 397.72 to 410.88 Mb, were significantly associated with RDW (Figure 4A). These five SNPs constituted a haploblock that can be divided into two haplotypes (*Hap1* and *Hap2*; Figure 4B). The frequency of the superior *Hap2* was 25.1% in the GWAS panel, showing that it has not been used in many modern wheat varieties. *Hap2* was proposed to significantly increase RDW at every stage, indicating its vital role in supporting root growth (Figure 4C). Furthermore, varieties harboring these elite alleles, including Yanke 028, Fengyou 6, Yamai 1, and Mengmai 023, contributed to 49.59 g of TKW on average, which was higher than that of other accessions (42.76 g). This region for RDW may be a promising locus to use and evaluate in future studies.

### Prediction of Candidate Genes for Wheat Root Traits

In this study, 12 co-localization regions on chromosomes 1D, 2A, 4A, 4B, 5B, 6D, and unmapped markers were found in wheat from different growing environments. Alignment to the reference wheat genome (Chinese Spring) revealed that three regions were located at similar positions or were consistent with those found in previous studies. In the 484.26–486.74 Mb region on chromosome 1D, which was 9 Mb from *Xbarc62_1D* (493.48 Mb) (Ibrahim et al., 2012; Sun et al., 2013), one and two SNPs were significantly associated with TRA under IHC and OHC, respectively. In the 657.45–658.78 Mb region on chromosome 4B, which was approximately 16 Mb from *Xwmc413_4B* (641.40 Mb), one SNP each in OHC and OPC was significantly associated with TRA. And *Xwmc413_4B* was associated with not only root area but also root length, root volume, and root dry weight (Maccaferri et al., 2016). Other pleiotropic SNPs associated with root traits were also detected in previous studies (Ren et al., 2012; Li et al., 2019). We predicted that these pleiotropic SNPs might contain tightly linked genes or encode transcription factors regulating multiple traits.

A co-localization region identified in all three growing environments regulating TRL was located within 737.85–742.00 Mb on chromosome 4A, which has not been reported. 27 candidate genes were detected in this interval, and four of these genes (*TraesCS4A02G484200*, *TraesCS4A02G484800*, *TraesCS4A02G493800*, and *TraesCS4A02G493900*) showed high expression levels and regulated root length based on their annotations and expression patterns. *TraesCS4A02G484200* encoded a protein containing zinc-finger-like metal binding domains (yippee domain). Zinc finger protein *OsCYP2* controlled lateral root development in response to auxin, whereas *OsCYP2* silencing leaded to significantly fewer lateral roots than in the wild-type rice plants (Cui et al., 2017). *TraesCS4A02G484100, TraesCS4A02G493800*, and *TraesCS4A02G494000*, which encode members of the F-box-like protein superfamily, were notably associated with E3 ubiquitin-mediated protein degradation in response to abiotic stress (Hua and Vierstra, 2011). *TaFBA1* encodes a homologous F-box protein in wheat that was translated into tobacco, and its overexpression improved heat tolerance and lengthened the root (Li et al., 2018). *TraesCS4A02G493900* and *TraesCS4A02G494100* encoded leucine-rich repeat domain, which was the important part of the leucine-rich repeat receptor-like kinase (LRR-RLK). Root growth is determined by meristem cell elongation and differentiation (Dello Ioio et al., 2007). One of the LRR-RLK genes contained 21 leucine-rich repeat domains, which were expressed in roots and rosettes of *Arabidopsis* and were involved in cellular signaling in plants (Walker, 1993). Another of the LRR-RLK genes (*OsSERK1*) with high expression levels was detected in rice calluses during somatic embryogenesis, and it promoted cell differentiation and mediated defense signal transduction (Hu et al., 2005). *TraesCS4A02G484800* and *TraesCS4A02G485000* encode the glycoside hydrolase family 32, leucine-rich repeat domain superfamily, and concanavalin A-like lectin/glucanase domain superfamily members. *At1G12240* in *Arabidopsis* encodes glycoside hydrolase family 32, which is involved in the decomposition and transformation of sucrose, which could promote hypocotyl elongation (Sergeeva et al., 2006). Similarly, *OsINV3* in rice is a gene that encodes glycoside hydrolase family proteins. Electron microscopy revealed that the panicles of *OsINV3* mutant plants displayed small and few cells on the inner and outer membranes, indicating that *OsINV3* plays an important role in cell expansion (Morey et al., 2018). *TraesCS4A02G484800* is homologous to *OsINV3* and *AtG12240*. These genes might regulate the elongation and formation of root cells through the zinc-finger domain, F-box-like proteins, leucine-rich repeat domains and glycoside hydrolases can thereby affecting root development.

## Conclusion

Correlation coefficients of root traits between OHC and OPC were higher than those for either IHC vs. OPC or IHC vs. OHC. And, RDW between OHC and OPC was closely correlated at earlier stages (seedling and wintering). Thus, OHC may be considered a rapid root identification method worthy of promotion. Among the 196 wheat accessions, Luomai 23, Zhongchuang 805, Yandian 9433, Kaimai 18, and Xinmai 19 had poor root systems, while Yumai 54, Luohan 6, Zhengpinmai 8, and Shangmai 156 had large root systems under all three growing environments. Root traits were negatively correlated with GY, SPA, and GPS, but positively correlated with TKW. 12 co-localization regions were detected with an *R*^2^ range of 6.38–19.43%. Among them, a stable region for TRL was detected within 737.85–742.00 Mb on chromosome 4A over three growing environments. In this region, there were 27 genes involved in cell elongation and differentiation, and some genes (*TraesCS4A02G484200, TraesCS4A02G484800*, *TraesCS4A02G493900*, and *TraesCS4A02G494000)* were highly expressed in root tissues. Another vital region on chromosome 5B may contribute to both RDW and TKW.

## Data Availability Statement

The raw data supporting the conclusions of this article will be made available by the authors, without undue reservation.

## Author Contributions

FX, KZ, and DH conceived the topic. SC and KZ provided gene chip. ZZ, YH, MS, and JZ collected the phenotype regulation data. SZ, XY, and JW performed the data analysis. FX and SC wrote the manuscript with edits from other co-authors. All authors read and approved the manuscript.

## Conflict of Interest

The authors declare that the research was conducted in the absence of any commercial or financial relationships that could be construed as a potential conflict of interest.

## Footnotes

1 http://wheat.pw.usda.gov/ggpages/topics/Wheat_660_SNP_array_developed_by_CAAS.pdf

2 https://github.com/YinLiLin/R-CMplot

3 http://plants.ensembl.org/hmmer/index.html

4 https://www.ncbi.nlm.nih.gov/nuccore/

5 https://wheat.pw.usda.gov/WheatExp/

## Supplementary Material

Supplementary Figure 1 Wheat under OPC growth at different stages.

Supplementary Figure 2 Plots of the frequency distributions of root traits under different growing environments and at different stages.

Supplementary Figure 3 Population structure analysis of the GWAS panel and LD decay. **(A)** Plot of delta K against putative K ranging from 1 to 10; **(B)** plot of first principle component against second principle components; **(C)** plot of clustering based on Archaeopteryx tree; **(D)** over different genetic distances (Mb) for the A,B,D subgenomes and whole hexaploid wheat genome.

Supplementary Figure 4 Manhattan and quantile-quantile (Q-Q) plots for root traits.

Supplementary Figure 5 Haploview plot for LD decay within the candidate region on chromosome 4A and TRL phenotypic effect of allele variation.

Supplementary Table 1 Information on the 196 wheat accessions, phenotypic and the corresponding *u*-values for root under different growing environments.

Supplementary Table 2 Difference of root traits among OHC, IHC, and OPC at seedling stage.

Supplementary Table 3 Correlation coefficients of root traits under different growing environments.

Supplementary Table 4 GWAS results for root traits according to the mixed linear model.

Supplementary Table 5 Genetic positions of SNPs in co-localization associated with root traits under different growing environments.

Supplementary Table 6 Physical positions and associated traits of the pleiotropic SNP markers from different growing environments.

Supplementary Table 7 Gene annotation of the genomic region (737.85–742.00 Mb) on chromosome 4A of Chinese Spring.

Supplementary Table 8 The expression data (fragments per kilobase of transcript per million reads sequenced, FPKM) in different organs at different stages.

## Abbreviations

TRL: Total length of roots
TRV: Total root volume
ARD: Average root diameter
TRA: Total root area
NRT: Number of root tips
RDW: Root dry weight
GY: Grain yield per area
SPA: Spikes per area
GPS: Grains per spike
TKW: Thousand-kernel weight
OHC: Outdoor hydroponic culture
IHC: Indoor hydroponic culture
OPC: Outdoor pot culture
SS: Seedling stage
WP: Wintering period
JS: Jointing stage
MS: Mature stage
GWAS: Genome-wide association study
BLUE: Best linear unbiased estimation
SNP: Single- nucleotide polymorphism
LD: Linkage disequilibrium
QTL: Quantitative trait loci.

## References

[B1] Atkinson J. A. Wingen L. U. Marcus G. Pound M. P. Oorbessy G. John F. M. (2015). Phenotyping pipeline reveals major seedling root growth QTL in hexaploid wheat. *J. Exp. Bot.* 66 2283–2292. 10.1093/jxb/erv006 25740921 PMC4407652

[B2] Atta B. M. Mahmood T. Trethowan R. M. (2013). Relationship between root morphology and grain yield of wheat in north-western NSW, Australia. *Aust. J. Crop Sci.* 7 2108–2115.

[B3] Ayalew H. Desalegn T. Hui L. Yan G. (2016). Performance of Ethiopian bread wheat (*Tritium aestivum* L.) genotypes under contrasting water regimes: Potential sources of variability for drought resistance breeding. *Aust. J. Crop Sci.* 10 370–376. 10.21475/ajcs.2016.10.03.p7230

[B4] Bai C. Ge Y. Ashton R. W. Evans J. Milne A. Hawkesford M. J. (2019). The relationships between seedling root screens, root growth in the field and grain yield for wheat. *Plant Soil.* 440 311–326. 10.1007/s11104-019-04088-9

[B5] Beyer S. Daba S. Tyagi P. Bockelman H. Brown-Guedira G. Mohammadi M. (2019). Loci and candidate genes controlling root traits in wheat seedlings—a wheat root GWAS. *Funct. Integr. Genomic.* 19 91–107. 10.1007/s10142-018-0630-z 30151724

[B6] Bishopp A. Lynch J. P. (2015). The hidden half of crop yields. *Nat. Plants* 1:1511. 10.1038/nplants.2015.117 27250548

[B7] Bradbury P. J. Zhang Z. Kroon D. E. Casstevens T. M. Ramdoss Y. Buckler E. S. (2007). TASSEL: software for association mapping of complex traits in diverse samples. *Bioinformatics* 23 2633–2635. 10.1093/bioinformatics/btm308 17586829

[B8] Cao P. Ren Y. Zhang K. Teng W. Zhao X. Dong Z. (2014). Further genetic analysis of a major quantitative trait locus controlling root length and related traits in common wheat. *Mol. Breeding* 33 975–985. 10.1007/s11032-013-0013-z

[B9] Chang H. Chen D. Kam J. Richardson T. Drenth J. Guo X. (2016). Abiotic stress upregulated TaZFP34 represses the expression of type-B response regulator and *SHY2* genes and enhances root to shoot ratio in wheat. *Plant Sci.* 252 88–102. 10.1016/j.plantsci.2016.07.011 27717481

[B10] Chen S. Cheng X. Yu K. Chang X. Bi H. Xu H. (2020). Genome-wide association study of differences in 14 agronomic traits under low- and high-density planting models based on the 660k SNP array for common wheat. *Plant Breed.* 139 272–283. 10.1111/pbr.12774

[B11] Christopher J. Christopher M. Jennings R. Jones S. Fletcher S. Borrell A. (2013). QTL for root angle and number in a population developed from bread wheats (*Triticum aestivum*) with contrasting adaptation to water-limited environments. *Theor. Appl. Genet.* 126 1563–1574. 10.1007/s00122-013-2074-0 23525632

[B12] Cui P. Liu H. Ruan S. Ali B. Gill R. A. Ma H. (2017). A zinc finger protein, interacted with cyclophilin, affects root development via IAA pathway in rice. *J. Integr. Plant Biol*. 59 496–505. 10.1111/jipb.12531 28267270

[B13] Dello Ioio R. Linhares F. S. Scacchi E. Casamitjana-Martinez E. Heidstra R. Costantino P. (2007). Cytokinins determine *Arabidopsis* root-meristem size by controlling cell differentiation. *Curr. Biol.* 17 678–782. 10.1016/j.cub.2007.02.047 17363254

[B14] Earl D. A. Vonholdt B. M. (2012). STRUCTURE HARVESTER: a website and program for visualizing STRUCTURE output and implementing the Evanno method. *Conserv. Genet. Resour.* 4 359–361. 10.1007/s12686-011-9548-7

[B15] Fang S. Yan X. Liao H. (2009). 3D reconstruction and dynamic modeling of root architecture in situ and its application to crop phosphorus research. *Plant J.* 60 1096–1108. 10.1111/j.1365-313X.2009.04009.x 19709387

[B16] Fang Y. Du Y. Wang J. Wu A. Qiao S. Xu B. (2017). Moderate drought stress affected root growth and grain yield in old, modern and newly released cultivars of winter wheat. *Front. Plant Sci.* 8:672. 10.3389/fpls.2017.00672 28507555 PMC5410596

[B17] González F. G. Miralles D. J. Slafer G. A. (2011). Wheat floret survival as related to pre-anthesis spike growth. *J. Exp. Bot.* 62 4889–4901. 10.1093/jxb/err182 21705386

[B18] Hu H. Xiong L. Yang Y. (2005). Rice SERK1 gene positively regulates somatic embryogenesis of cultured cell and host defense response against fungal infection. *Planta* 222 107–117. 10.1007/s00425-005-1534-4 15968510

[B19] Hua Z. Vierstra R. D. (2011). The cullin-ring ubiquitin-protein ligases. *Annu. Rev. Plant Biol.* 62 299–334. 10.1146/annurev-arplant-042809-112256 21370976

[B20] Ibrahim S. E. Schubert A. Pillen K. Léon J. (2012). QTL analysis of drought tolerance for seedling root morphological traits in an advanced backcross population of spring wheat. *Int. J. Agrc. Stat. Sci.* 2 619–629.

[B21] Jakobsson M. Rosenberg N. A. (2007). CLUMPP: a cluster matching and permutation program for dealing with label switching and multimodality in analysis of population structure. *Bioinformatics* 23 1801–1806. 10.1093/bioinformatics/btm233 17485429

[B22] Kabir M. R. Liu G. Guan P. Wang F. Khan A. A. Ni Z. (2015). Mapping QTLs associated with root traits using two different populations in wheat (*Triticum aestivum* L.). *Euphytica* 206 175–190. 10.1007/s10681-015-1495-z

[B23] Lei M. Li H. Zhang L. Wang J. (2015). QTL IciMapping: Integrated software for genetic linkage map construction and quantitative trait locus mapping in biparental populations. *Crop J.* 3 269–283. 10.1016/j.cj.2015.01.001

[B24] Li B. Liu D. Li Q. Mao X. Li A. Wang J. (2016). Overexpression of wheat gene *TaMOR* improves root system architecture and grain yield in *Oryza sativa*. *J. Exp. Bot.* 67 4155–4167. 10.1093/jxb/erw193 27229732 PMC5301925

[B25] Li L. Peng Z. Mao X. Wang J. Chang X. Reynolds M. (2019). Genome-wide association study reveals genomic regions controlling root and shoot traits at late growth stages in wheat. *Ann. Bot.* 124:6. 10.1093/aob/mcz041 31329816 PMC6881226

[B26] Li Q. Wang W. Wang W. Zhang G. Liu Y. Wang Y. (2018). Wheat F-box protein gene *TaFBA1* is involved in plant tolerance to heat stress. *Front. Plant Sci*. 9:521. 10.3389/fpls.2018.00521 29740462 PMC5928245

[B27] Liu X. Li R. Chang X. Jing R. (2013). Mapping QTLs for seedling root traits in a doubled haploid wheat population under different water regimes. *Euphytica* 189 51–66. 10.1007/s10681-012-0690-4

[B28] Liu Y. Wang L. Mao S. Liu K. Zheng Y. (2015). Genome-wide association study of 29 morphological traits in *Aegilops tauschii*. *Sci. Rep.* 5 15562. 10.1038/srep15562 26503608 PMC4622089

[B29] Liu Y. Lin Y. Gao S. Li Z. Ma J. Deng M. (2017). A genome-wide association study of 23 agronomic traits in chinese wheat landraces. *Plant J.* 91:861. 10.1111/tpj.13614 28628238

[B30] Lopes M. S. Reynolds M. P. (2010). Partitioning of assimilates to deeper roots is associated with cooler canopies and increased yield under drought in wheat. *Funct. Plant Biol*. 37 147–156. 10.1071/FP09121

[B31] Ma S. C. Xu B. C. Li F. M. Liu W. Z. Huang Z. B. (2008). Effects of root pruning on competitive ability and water use efficiency in winter wheat. *Field Crop Res*. 105 56–63. 10.1016/j.fcr.2007.07.005

[B32] Maccaferri M. El-Feki W. Nazemi G. Salvi S. Canè M. A. Colalongo M. C. (2016). Prioritizing quantitative trait loci for root system architecture in tetraploid wheat. *J. Exp. Bot.* 67 1161–1178. 10.1093/jxb/erw039 26880749 PMC4753857

[B33] Man J. Shi Y. Yu Z. Zhang Y. (2015). Dry matter production, photosynthesis of flag leaves and water use in winter wheat are affected by supplemental irrigation in the Huang-Huai-Hai plain of China. *PLoS One* 10:e0137274. 10.1371/journal.pone.0137274 26335019 PMC4559388

[B34] Morey S. R. Hirose T. Hashida Y. Miyao A. Hirochika H. Ohsugi R. (2018). Genetic evidence for the role of a rice vacuolar invertase as a molecular sink strength determinant. *Rice* 11:6. 10.1186/s12284-018-0201-x 29344835 PMC5772344

[B35] Neumann G. George T. S. Plassard C. (2009). Strategies and methods for studying the rhizosphere—the plant science toolbox. *Plant Soil.* 321 431–456. 10.1007/s11104-009-9953-9

[B36] Neumann K. Kobiljski B. Deni S. Varshney R. K. Brner A. (2011). Genome-wide association mapping: a case study in bread wheat (*Triticum aestivum* L.). *Mol. Breed.* 27 37–58. 10.1007/s11032-010-9411-7

[B37] Osmont K. S. Sibout R. Hardtke C. S. (2007). Hidden branches: developments in root system architecture. *Annu. Rev. Plant Biol.* 58 93–113. 10.1146/annurev.arplant.58.032806.104006 17177637

[B38] Passioura J. B. (1983). Roots and drought resistance. *Agr. Water Manage.* 7 265–280. 10.1016/0378-3774(83)90089-6

[B39] Ren Y. He X. Liu D. Li J. Zhao X. Li B. (2012). Major quantitative trait loci for seminal root morphology of wheat seedlings. *Mol. Breed.* 30 139–148. 10.1007/s11032-011-9605-7

[B40] Reynolds M. P. Pierre C. S. Saad A. S. Vargas M. Condon A. G. (2007). Evaluating potential genetic gains in wheat associated with stressadaptive trait expression in elite genetic resources under drought and heat stress. *Crop Sci*. 47 172–189. 10.2135/cropsci2007.10.002

[B41] Robertson B. M. Waines J. G. Gill B. S. (1979). Genetic Variability for Seedling Root Numbers in Wild and Domesticated Wheats1. *Crop Sci.* 19 843–847. 10.2135/cropsci1979.0011183X001900060024x

[B42] Sergeeva L. I. Keurentjes J. J. Bentsink L. Vonk J. van der Plas L. H. Koornneef M. (2006). Vacuolar invertase regulates elongation of *Arabidopsis thaliana* roots as revealed by QTL and mutant analysis. *Proc. Natl. Acad. Sci. USA.* 103 2994–2999. 10.1073/pnas.0511015103 16481625 PMC1413815

[B43] Steele K. A. Price A. H. Witcombe J. R. Shrestha R. Singh B. N. Gibbons J. M. (2013). QTLs associated with root traits increase yield in upland rice when transferred through marker-assisted selection. *Theor. Appl. Genet.* 126 101–108. 10.1007/s00122-012-1963-y 22968512

[B44] Steinemann S. Zeng Z. H. McKay A. Heuer S. Langridge P. Huang C. Y. (2015). Dynamic root responses to drought and rewatering in two wheat (*Triticum aestivum*) genotypes. *Plant Soil.* 391 139–152. 10.1007/s11104-015-2413-9

[B45] Sukumaran S. Dreisigacker S. Lopes M. Chavez P. Reynolds M. P. (2015). Genome-wide association study for grain yield and related traits in an elite spring wheat population grown in temperate irrigated environments. *Theor. Appl. Genet.* 128 353–363. 10.1007/s00122-014-2435-3 25490985

[B46] Sun J. J. Guo Y. Zhang G. Z. Gao M. G. Li S. S. (2013). QTL mapping for seedling traits under different nitrogen forms in wheat. *Euphytica* 191 317–331. 10.1007/s10681-012-0834-6

[B47] Suraj G. Sujan M. Michael B. J. Mai X. Gina B. G. Adhikari T. B. (2014). Genome-wide association study reveals novel quantitative trait loci associated with resistance to multiple leaf spot diseases of spring wheat. *PLoS One* 9:e108179. 10.1371/journal.pone.0108179 25268502 PMC4182470

[B48] Valassi A. Chierici R. (2014). Information and treatment of unknown correlations in the combination of measurements using the BLUE method. *Eur. Phys. J. C.* 74:2717. 10.1140/epjc/s10052-014-2717-6

[B49] Wang S. X. Zhu Y. L. Zhang D. X. Shao H. Ma C. X. (2017). Genome-wide association study for grain yield and related traits in elite wheat varieties and advanced lines using snp markers. *PLoS One* 12:e0188662. 10.1371/journal.pone.0188662 29176820 PMC5703539

[B50] Walker J. C. (1993). Receptor-like protein kinase genes of *Arabidopsis thaliana*. *Plant J.* 3 451–456. 10.1111/j.1365-313x.1993.tb00164.x 8220453

[B51] Xie Q. Fernando K. M. C. Mayes S. Sparkes D. L. (2017). Identifying seedling root architectural traits associated with yield and yield components in wheat. *Ann. Bot.* 119 1115–1129. 10.1093/aob/mcx001 28200109 PMC5604548

[B52] Ye X. Li J. Cheng Y. Yao F. Chen G. (2019). Genome-wide association study reveals new loci for yield-related traits in Sichuan wheat germplasm under stripe rust stress. *BMC Genomics* 20:640. 10.1186/s12864-019-6005-6 31395029 PMC6688255

[B53] Zheng X. Wen X. Qiao L. Zhao J. Zhang X. Li X. (2019). A novel QTL *QTrl.saw-2D.2* associated with the total root length identified by linkage and association analyses in wheat (*Triticum aestivum* L.). *Planta* 250 129–143. 10.1007/s00425-019-0315 30944981

