# Supplementary material for: Genome-Wide Association Study on Root Traits Under Different Growing Environments in Wheat (Triticum aestivum L.)
Source: Front Genet. 2021 Jun 10;12:646712. doi: 10.3389/fgene.2021.646712 (PMC8222912; doi:10.3389/fgene.2021.646712)
Supplement: Supplementary Figure 1 — Wheat under OPC growth at different stages. [file Image_1.pdf]

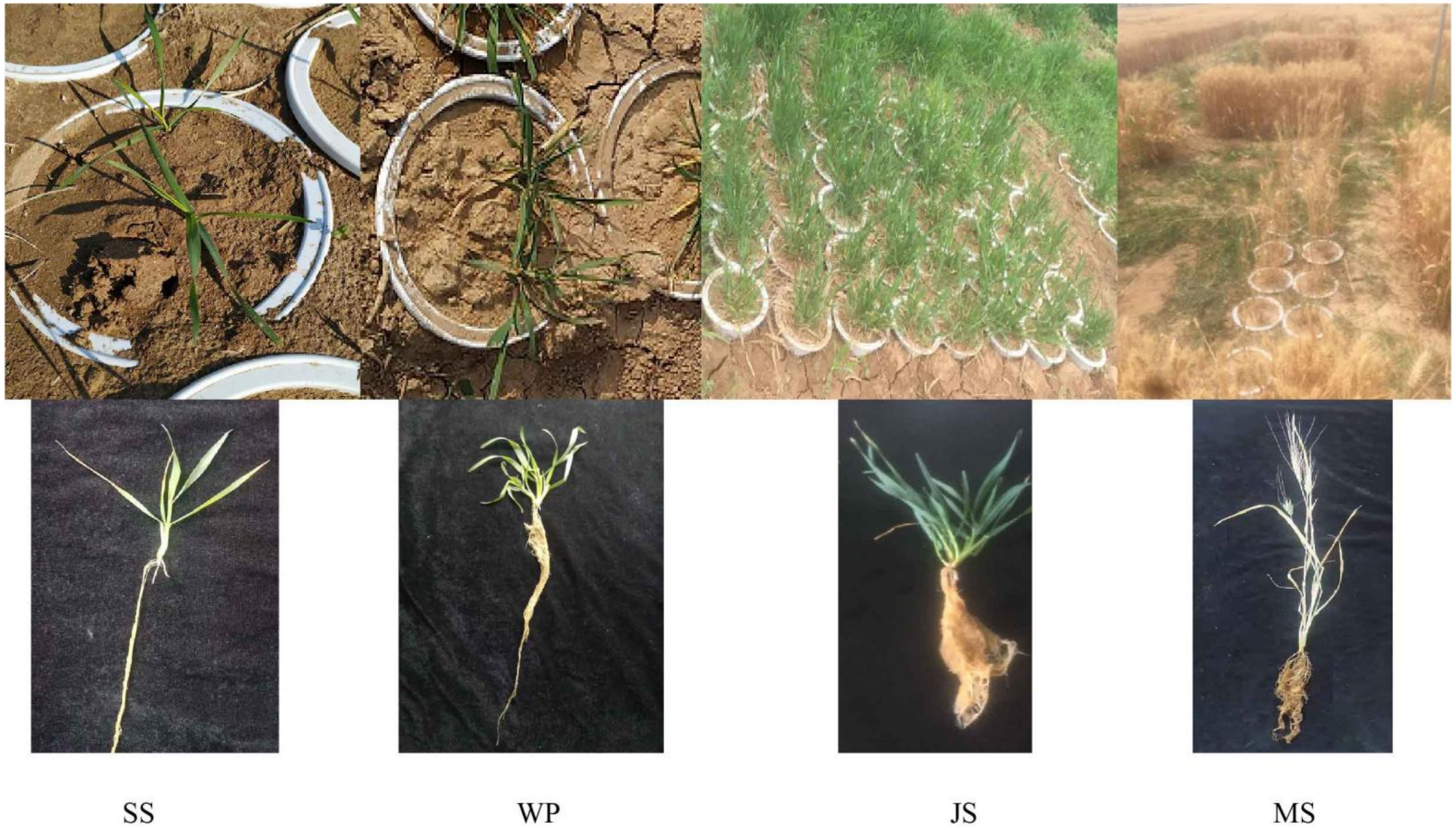

FIGURE S1 Wheat under OPC growth at different stages. SS, seedling stage; WP, wintering period; JS, jointing stage; MS, mature stage.
