# Supplementary material for: Genome-Wide Association Study on Root Traits Under Different Growing Environments in Wheat (Triticum aestivum L.)
Source: Front Genet. 2021 Jun 10;12:646712. doi: 10.3389/fgene.2021.646712 (PMC8222912; doi:10.3389/fgene.2021.646712)
Supplement: Supplementary Figure 2 — Plots of the frequency distributions of root traits under different growing environments and at different stages. [file Image_2.pdf]

A

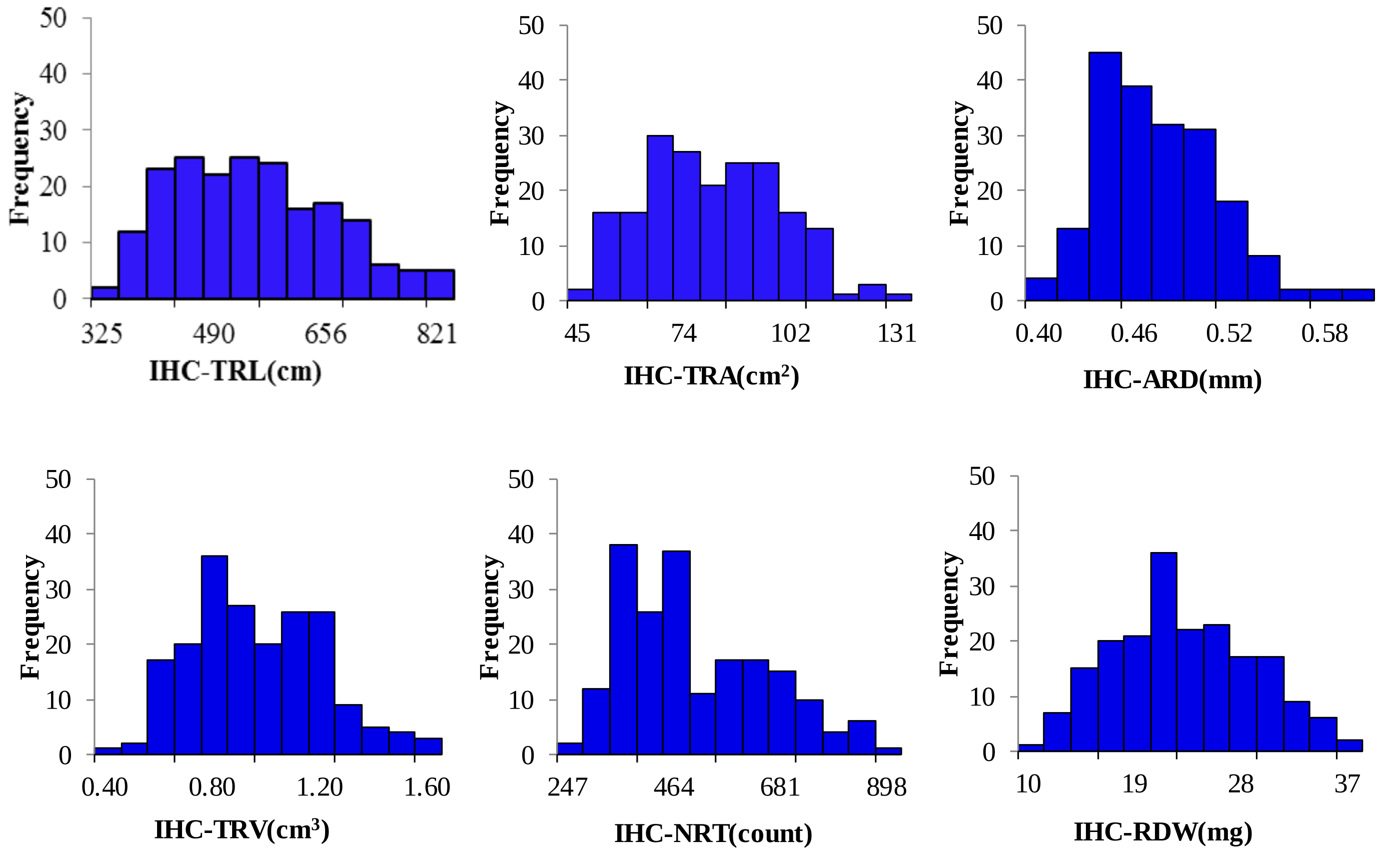

Frequency
IHC-TRL(cm)
IHC-TRA(cm2)
IHC-ARD(mm)
IHC-TRV(cm3)
IHC-NRT(count)
IHC-RDW(mg)

B

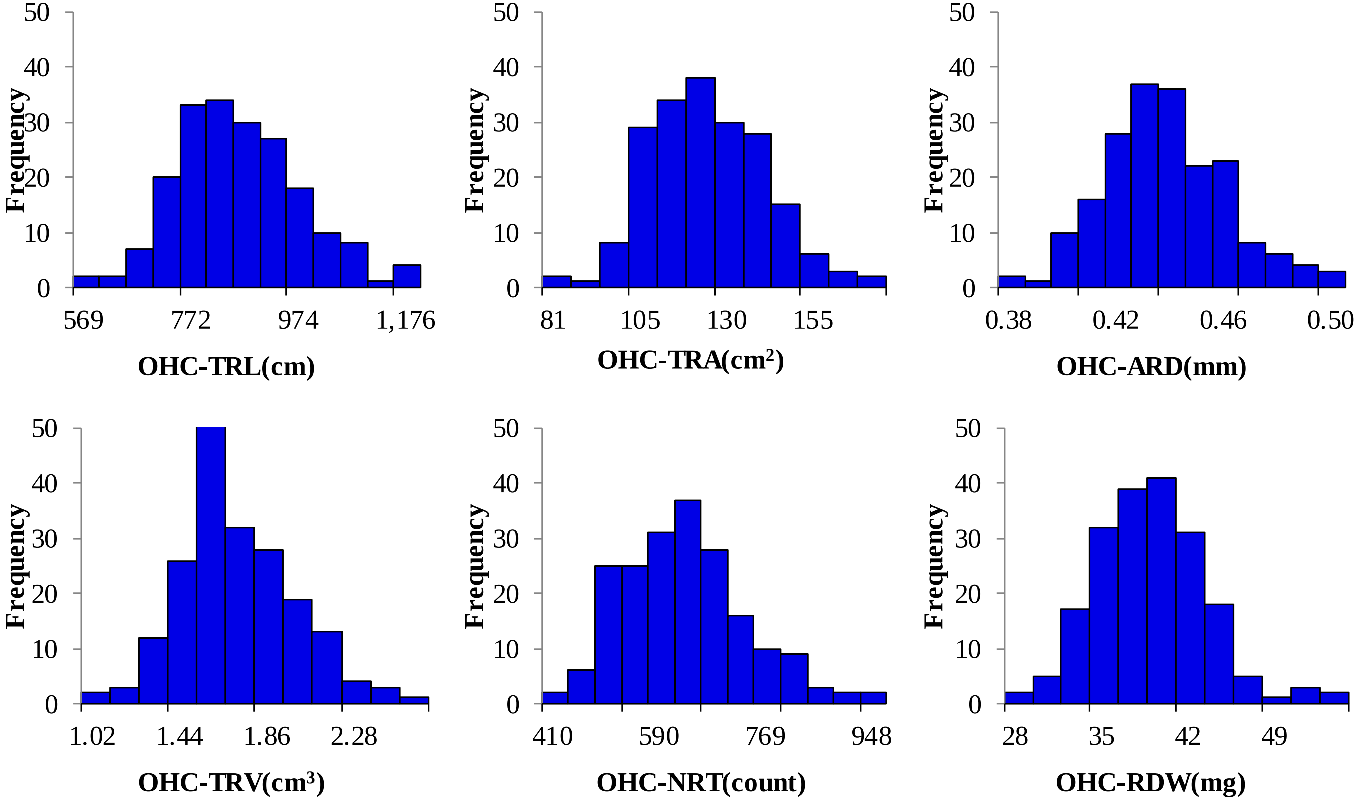

Frequency
OHC-TRL(cm)
569
772
974
1,176
OHC-TRA(cm²)
81
105
130
155
OHC-ARD(mm)
0.38
0.42
0.46
0.50
OHC-TRV(cm³)
1.02
1.44
1.86
2.28
OHC-NRT(count)
410
590
769
948
OHC-RDW(mg)
28
35
42
49
0
10
20
30
40
50

C

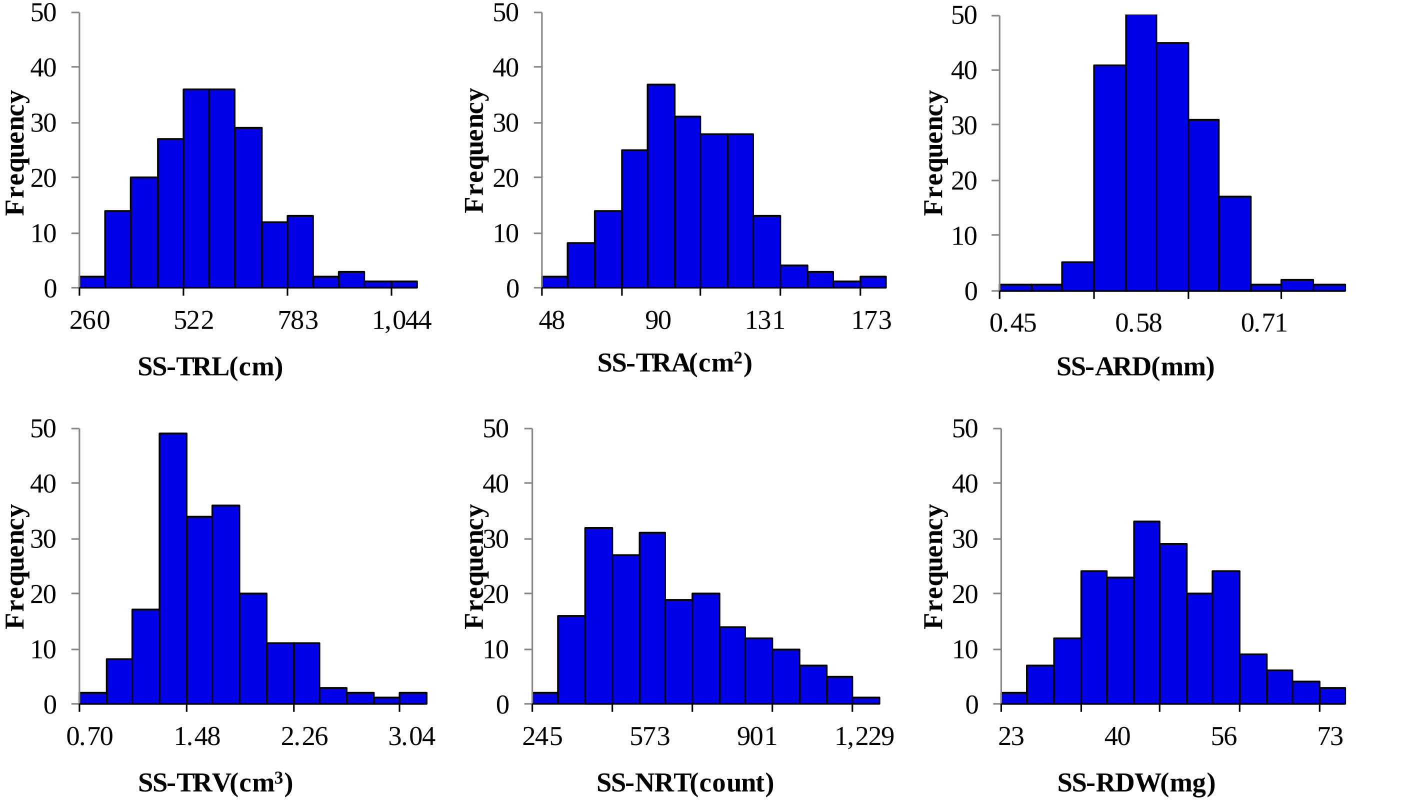

Frequency
50
40
30
20
10
0
260
522
783
1,044
SS-TRL(cm)
48
90
131
173
SS-TRA(cm²)
0.45
0.58
0.71
SS-ARD(mm)
0.70
1.48
2.26
3.04
SS-TRV(cm³)
245
573
901
1,229
SS-NRT(count)
23
40
56
73
SS-RDW(mg)

D

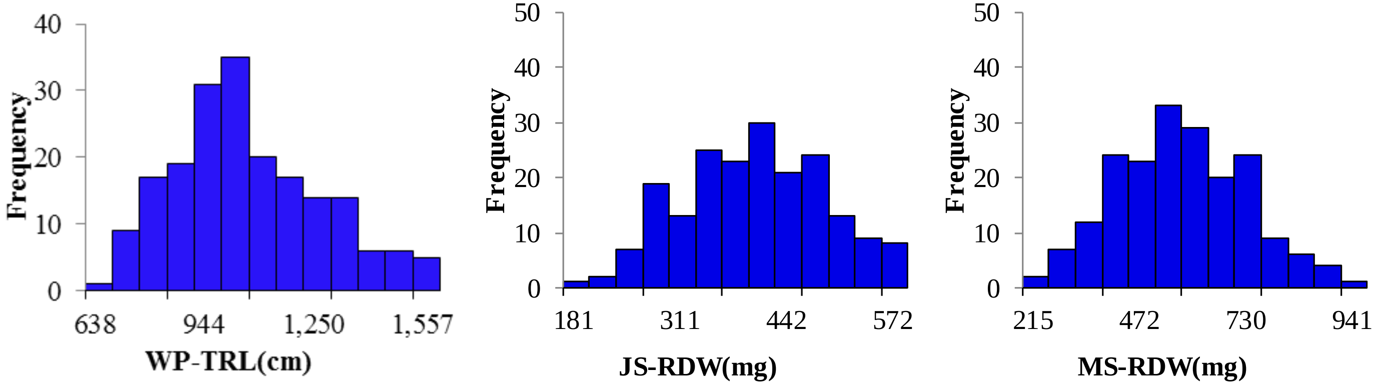

FIFURE S2 Plots of frequency distributions of root traits under different culture patterns and stages. a, IHC; b, OHC; c, SS; d, RDW TRL, total root length; TRV, total root volume; NRT, number of root tips; TRA, total root area; ARD, average root diameter; RDW, root dry weight; IHC, indoor hydroponic culture; OHC, outdoor hydro ponic culture; OPC, outdoor pot culture; SS, seedling stage; WP, wintering period; JS, jointing stage; MS, mature stage.
