# Supplementary material for: Genome-Wide Association Study on Root Traits Under Different Growing Environments in Wheat (Triticum aestivum L.)
Source: Front Genet. 2021 Jun 10;12:646712. doi: 10.3389/fgene.2021.646712 (PMC8222912; doi:10.3389/fgene.2021.646712)
Supplement: Supplementary Figure 3 — Population structure analysis of the GWAS panel and LD decay. (A) Plot of delta K against putative K ranging from 1 to 10; (B) plot of first principle component against second principle components; (C) plot of clustering based on Archaeopteryx tree; (D) over different genetic distances (Mb) for the A,B,D subgenomes and whole hexaploid wheat genome. [file Image_3.pdf]

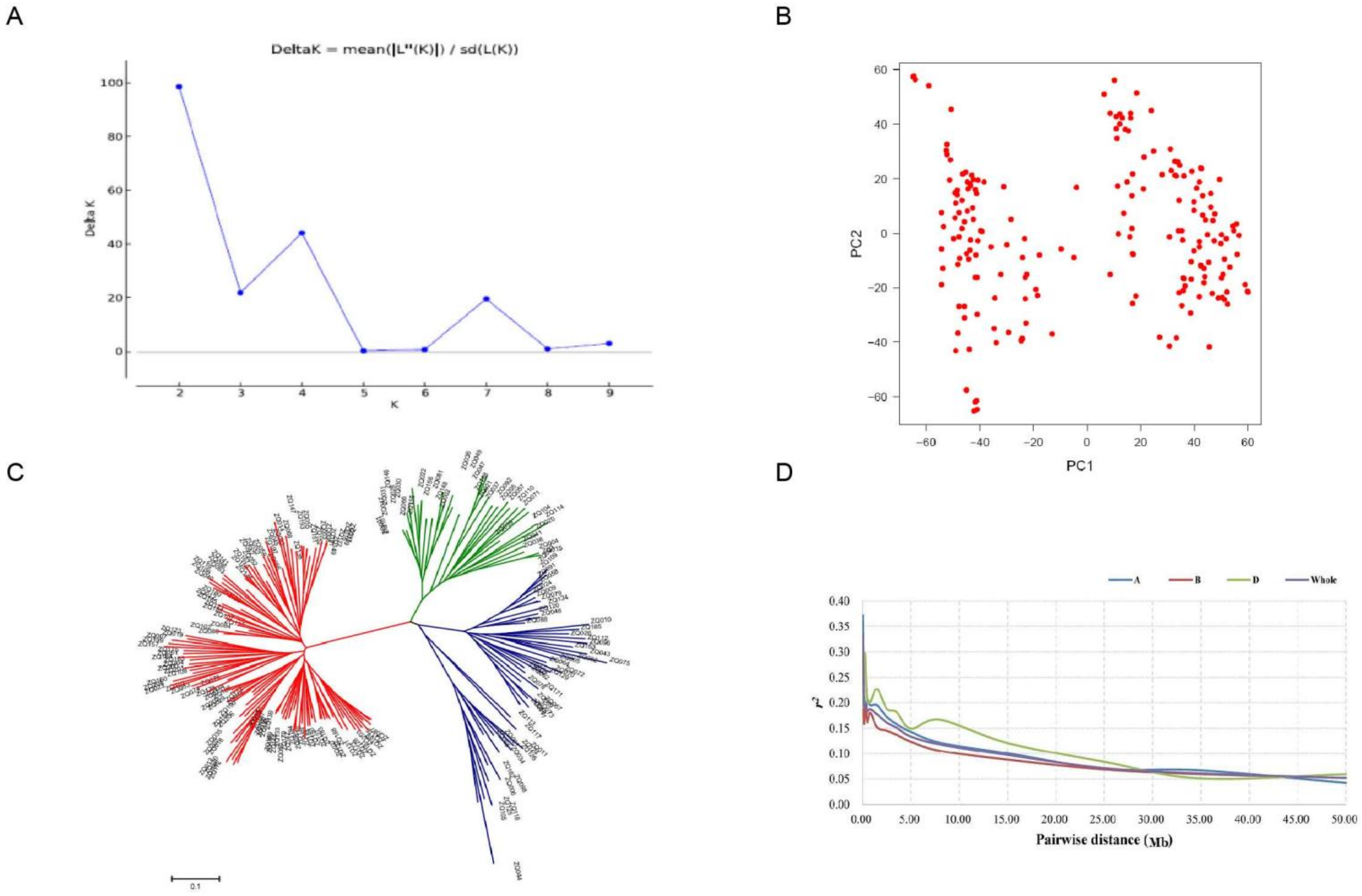

Figure S3 Population structure analysis of the GWAS panel and LD decay. (a) Plot of delta K against putative K ranging from 1 to 10; (b) plot of first principle component against second principle components; (c) plot of clustering based on Archaeopteryx tree; (d) over different genetic distances (Mb) for the A, B and D subgenomes and whole hexaploid wheat genome.
