# Supplementary material for: Genome-Wide Association Study on Root Traits Under Different Growing Environments in Wheat (Triticum aestivum L.)
Source: Front Genet. 2021 Jun 10;12:646712. doi: 10.3389/fgene.2021.646712 (PMC8222912; doi:10.3389/fgene.2021.646712)
Supplement: Supplementary Figure 4 — Manhattan and quantile-quantile (Q-Q) plots for root traits. [file Image_4.pdf]

IHC-TRL

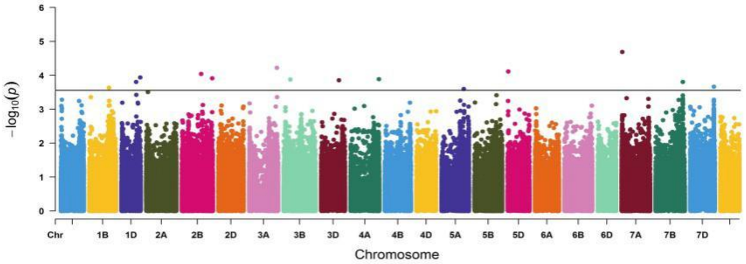

IHC-TRL

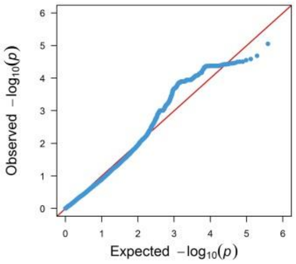

IHC-TRA

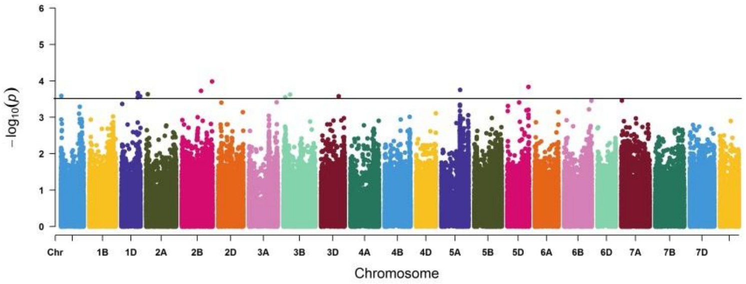

IHC-TRA

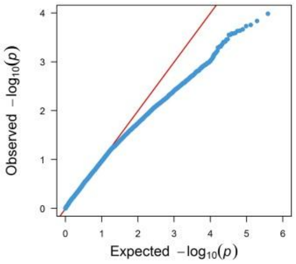

IHC-ARD

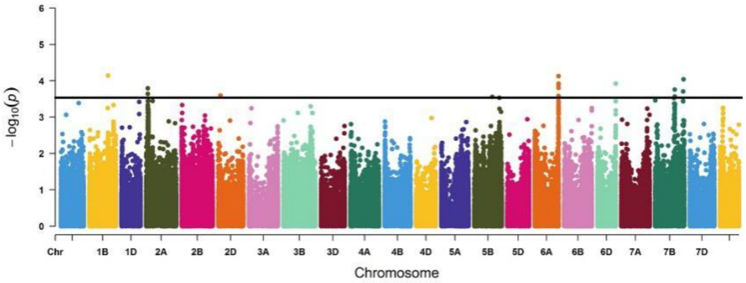

IHC-ARD

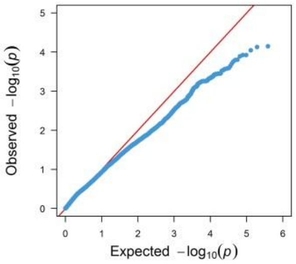

IHC-TRV

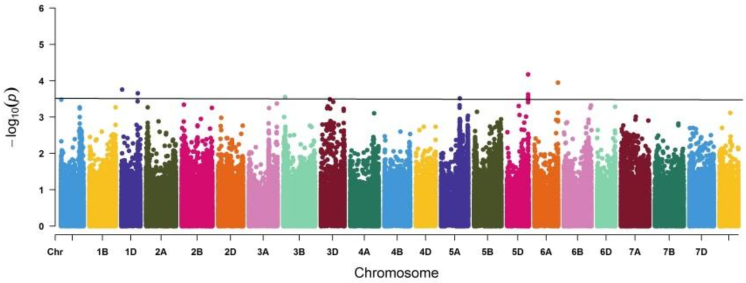

IHC-TRV

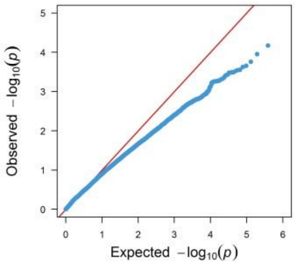

IHC-NRT

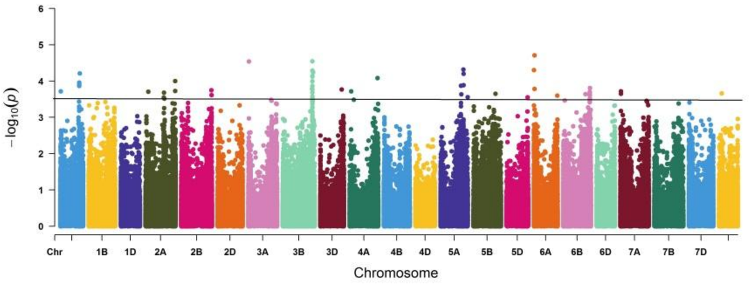

IHC-NRT

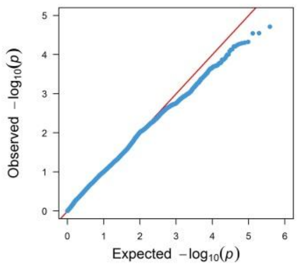

IHC-RDW

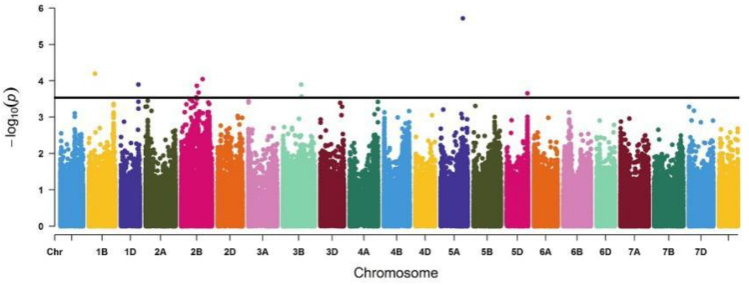

IHC-RDW

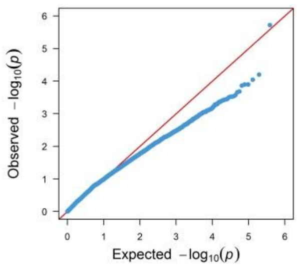

FIGURE S4-1 Manhattan and QQ-plots for six root traits in indoor hydroponic culture (IHC).

OHC-TRL

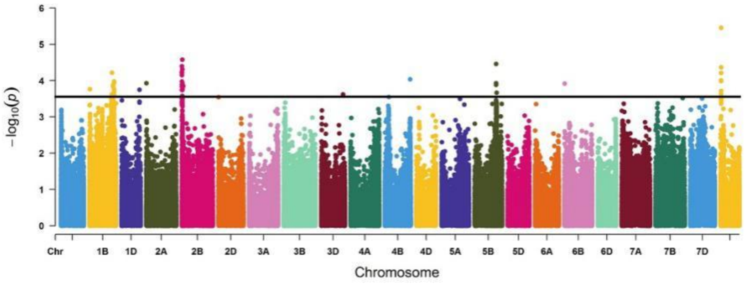

OHC-TRL

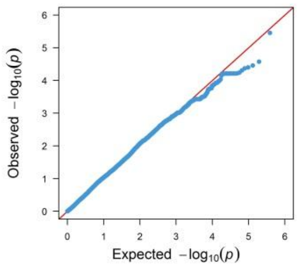

OHC-TRA

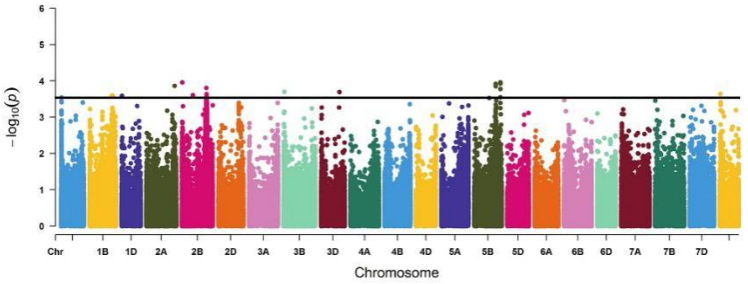

OHC-TRA

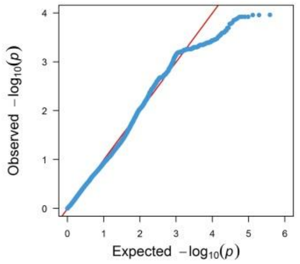

OHC-ARD

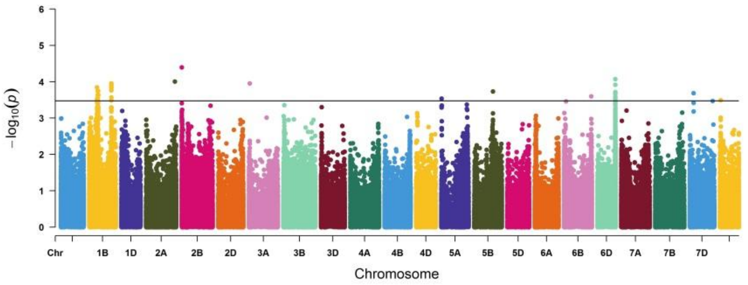

OHC-ARD

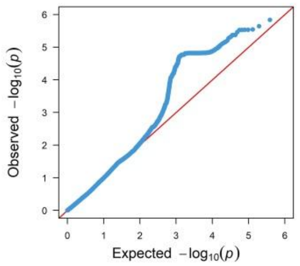

OHC-TRV

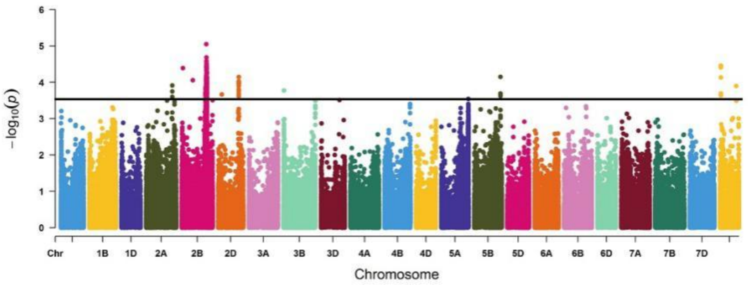

OHC-TRV

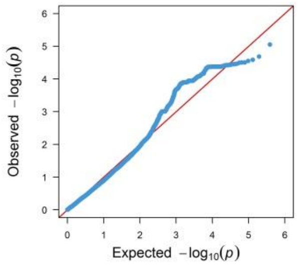

OHC-NRT OHC-NRT

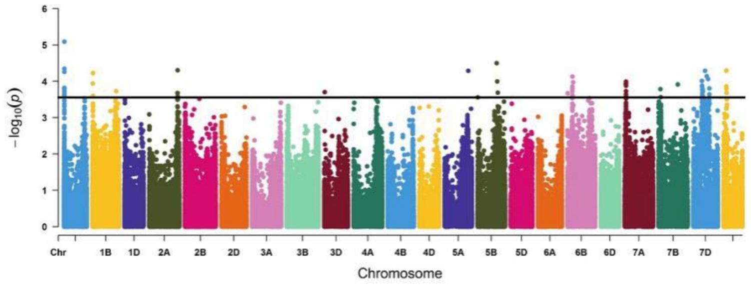

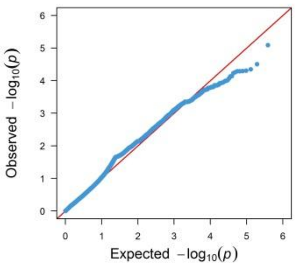

OHC-RDW OHC-RDW

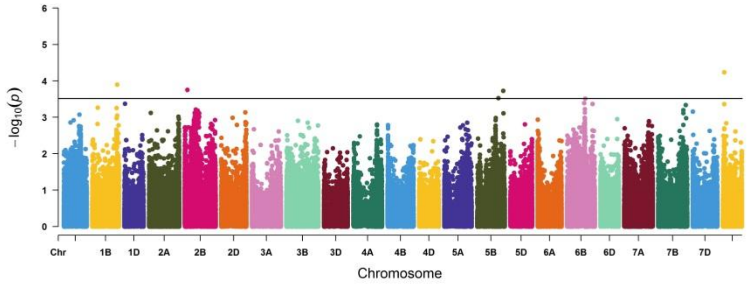

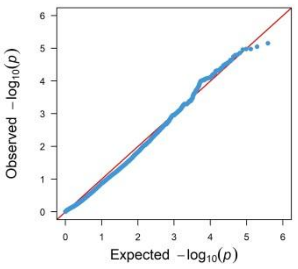

FIGURE S4-2 Manhattan and QQ-plots for 6 root traits in outdoor hydroponic culture (OHC).

OPC-TRL

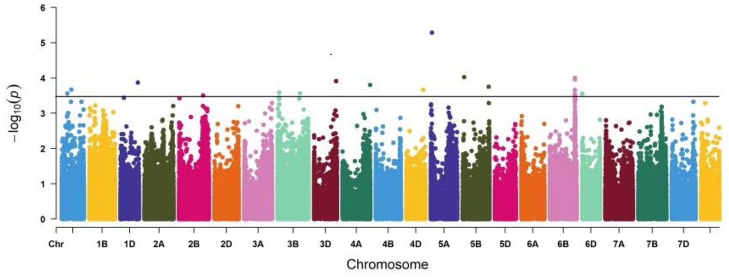

OPC-TRL

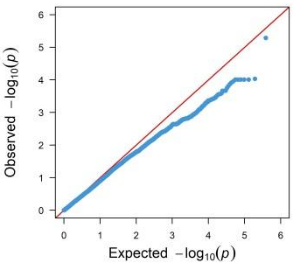

OPC-TRA

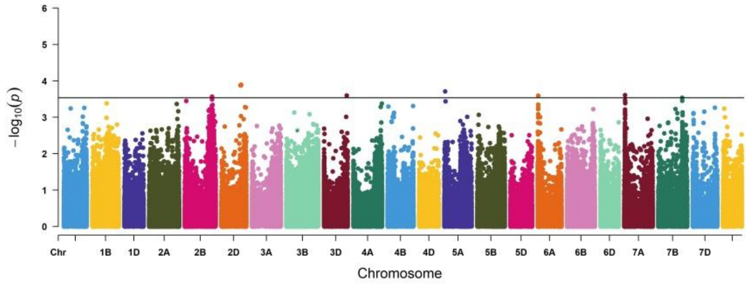

OPC-TRA

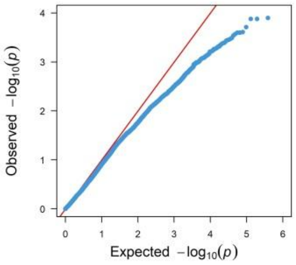

OPC-ARD

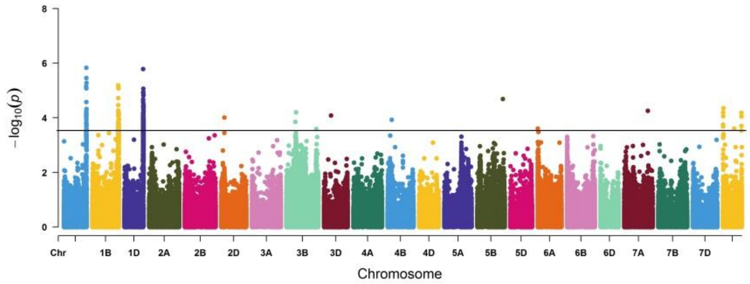

OPC-ARD

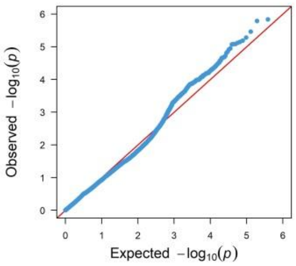

OPC-TRV

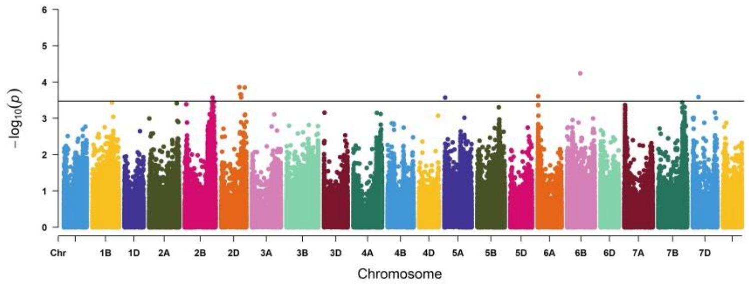

OPC-TRV

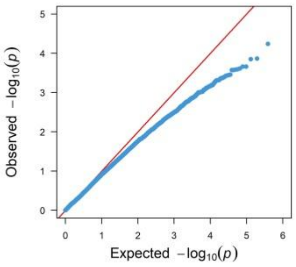

OPC-NRT OPC-NRT

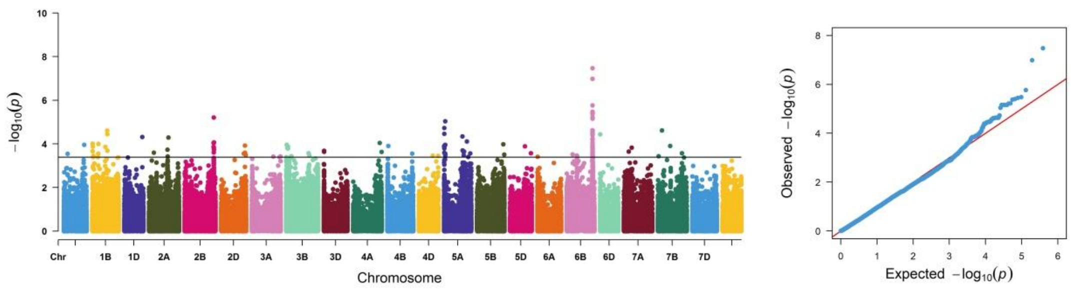

OPC-RDW OPC-RDW

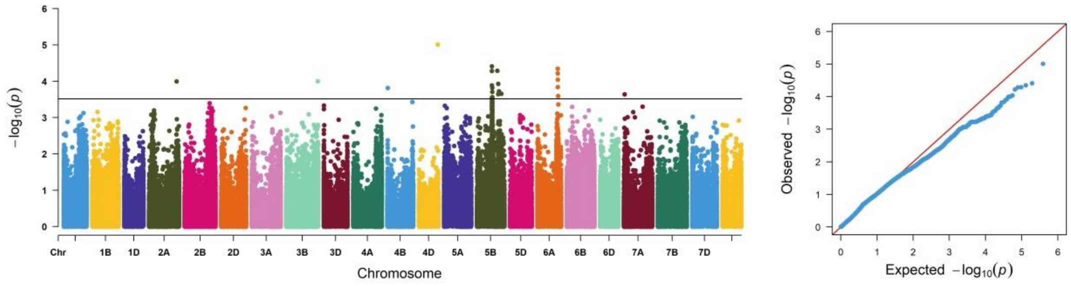

FIGURE S4-3 Manhattan and QQ-plots for root traits under outdoor pot culture (OPC).
