# Supplementary material for: Genome-Wide Association Study on Root Traits Under Different Growing Environments in Wheat (Triticum aestivum L.)
Source: Front Genet. 2021 Jun 10;12:646712. doi: 10.3389/fgene.2021.646712 (PMC8222912; doi:10.3389/fgene.2021.646712)
Supplement: Supplementary Figure 5 — Haploview plot for LD decay within the candidate region on chromosome 4A and TRL phenotypic effect of allele variation. [file Image_5.pdf]

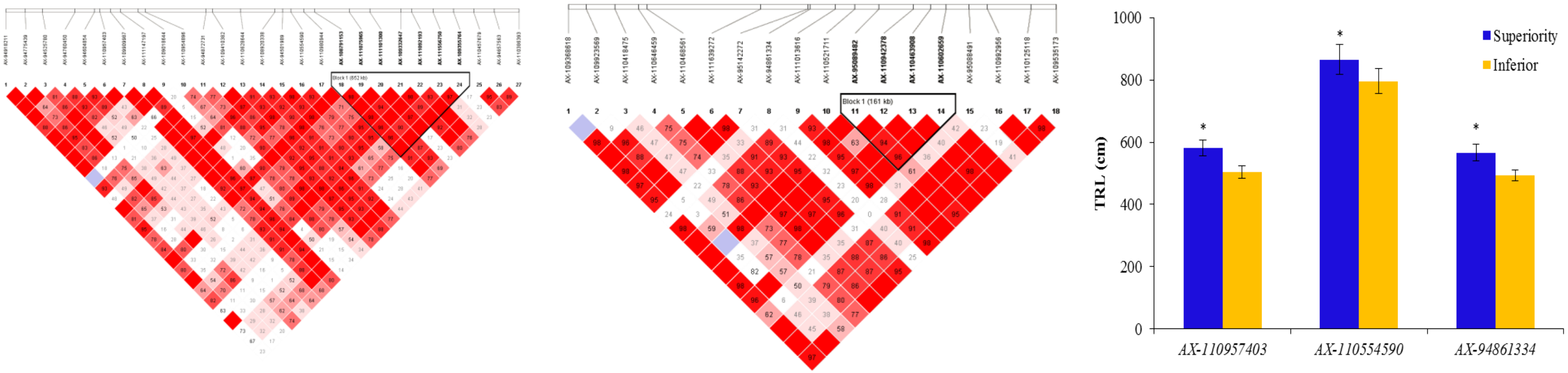

A **B** **C**

FIGURE S5 Two haploblocks for local LD decay within the candidate region on chromosome 4A for TRL and phenotypic effect of allele variation.
